# Physiological Biochemistry-Combined Transcriptomic Analysis Reveals Mechanism of *Bacillus cereus* G2 Improved Salt-Stress Tolerance of *Glycyrrhiza uralensis* Fisch. Seedlings by Balancing Carbohydrate Metabolism

**DOI:** 10.3389/fpls.2021.712363

**Published:** 2022-01-04

**Authors:** Xiang Xiao, Qiuli Wang, Xin Ma, Duoyong Lang, Zhenggang Guo, Xinhui Zhang

**Affiliations:** ^1^College of Pharmacy, Ningxia Medical University, Yinchuan, China; ^2^Laboratory Animal Center, Ningxia Medical University, Yinchuan, China; ^3^College of Pastoral Agriculture Science and Technology, Lanzhou University, Lanzhou, China; ^4^Ningxia Engineering and Technology Research Center of Hui Medicine Modernization, Ningxia Collaborative Innovation Center of Hui Medicine, Key Laboratory of Ningxia Minority Medicine Modernization, Ministry of Education, Ningxia Medical University, Yinchuan, China

**Keywords:** salt stress, *Bacillus cereus* G2, photosynthesis, carbohydrate transformation, glycolysis, tricarboxylic acid cycle

## Abstract

Salt stress severely threatens the growth and productivity of *Glycyrrhiza uralensis*. Previous results found that *Bacillus cereus* G2 enhanced several carbohydrate contents in *G. uralensis* under salt stress. Here, we analyzed the changes in parameters related to growth, photosynthesis, carbohydrate transformation, and the glycolysis Embden-Meyerhof-Parnas (EMP) pathway-tricarboxylic acid (TCA) cycle by G2 in *G. uralensis* under salt stress. Results showed that G2 helped *G. uralensis*-accumulating photosynthetic pigments during photosynthesis, which could further increase starch, sucrose, and fructose contents during carbohydrate transformation. Specifically, increased soluble starch synthase (SSS) activity caused to higher starch content, which could induce α-amylase (AM) and β-amylase (BM) activities; increased sucrose content due to the increase of sucrose synthase (SS) activity through upregulating the gene-encoding SS, which decreased cell osmotic potential, and consequently, induced invertase and gene-encoding α-glucosidase that decomposed sucrose to fructose, ultimately avoided further water loss; increased fructose content-required highly hexokinase (HK) activity to phosphorylate in *G. uralensis*, thereby providing sufficient substrate for EMP. However, G2 decreased phosphofructokinase (PFK) and pyruvate kinase (PK) activities during EMP. For inducing the TCA cycle to produce more energy, G2 increased PDH activity that enhanced CA content, which further increased isocitrate dehydrogenase (ICDH) activity and provided intermediate products for the *G. uralensis* TCA cycle under salt stress. In sum, G2 could improve photosynthetic efficiency and carbohydrate transformation to enhance carbohydrate products, thereby releasing more chemical energy stored in carbohydrates through the EMP pathway-TCA cycle, finally maintain normal life activities, and promote the growth of *G. uralensis* under salt stress.

## Introduction

In plants, salt stress leads to reduced water uptake, excessive accumulation of toxic elemental ions, and production of reactive oxygen species (ROS) causing oxidative stress (Salimi et al., 2016; Ahmadi et al., 2018). This combination of osmotic, ionic, and oxidative effects promotes cellular damage, declines K^+^ and Ca^2+^ efficiency, reduces photosynthetic rate, impairs metabolism, and ultimately inhibits plant growth and reduces productivity (Salimi et al., 2016; Brahimova et al., 2021). In response to salt-stressed condition, carbohydrate accumulation can be an important part of radical scavenging, osmo-protection, and carbon storage in plants (Keunen et al., 2013; Zhu et al., 2016; Li et al., 2020). Carbohydrates, as the products of photosynthesis, are not only the substrates for respiration but also important substances related to the growth and energy metabolism in plants (Raessler et al., 2010). Salt stress directly reduced CO_2_ availability through diffusion limitation of stomata and mesophyll or change photosynthetic metabolism, and also affected oxidative stress and thus indirectly affected leaf photosynthesis (Chaves et al., 2008). Some studies showed that salt stress typically reduced the chlorophyll (Chl) content in plants (Khoshbakht et al., 2015; Ibrahim et al., 2017), while other studies reported increased Chl concentration with increasing salinity stress in salt-tolerant plants (Borghesi et al., 2011; Huang et al., 2015; Qiu et al., 2017). Salt stress caused inhibition of carbon assimilation by affecting the activities of carbon metabolism-related enzymes and the contents of carbohydrate (Li et al., 2020). The glycolysis (EMP) pathway-tricarboxylic acid (TCA) cycle serves as the primary pathway for carbohydrate catabolism, providing sufficient energy for the plant’s life activities (Jiang, 2011). Moreover, salt stress led to the disorder of EMP metabolism, which posed a threat to plant respiration metabolism (Zhong et al., 2016). Salt stress also inhibited the TCA cycle, followed by a significant decrease in adenosine triphosphate (ATP) content, resulting in insufficient energy supply and further inhibition of plants’ growth (Li et al., 2015, 2020). Thus, it can be concluded that plants exposed to salt stress are unable to maintain the required carbohydrate and energy levels, and, consequently, certain measures must be adopted to regulate carbohydrate metabolism in salt-stressed plants.

Plant endophytes have great potential to enhance plant growth and alleviate salt stress without harming the environment (Fan et al., 2020; Molina-Montenegro et al., 2020). Previous studies revealed that endophytes increased salt tolerance of plants by regulating carbohydrate metabolism (Ali et al., 2014; Ghaffari et al., 2016; Win et al., 2018). Specifically, wild-type bacterial endophytes significantly increased the total Chl contents of tomato plants under salt stress (Ali et al., 2014). Endophyte (*Penicillium funiculosum* LHL06) significantly increased Chl contents and the photosynthesis rate of *Glycine max* L. under salt stress (Khan et al., 2011). Moreover, the endophytic fungus *Piriformospora indica* increased the starch concentration in barley plants under salt stress (Ghaffari et al., 2016). Therefore, carbohydrate regulation may be one of the important strategies for improving plant salt tolerance by endophytes. However, the deep mechanisms of endophytes on carbohydrate regulation in plants exposed to salt stress are largely unknown.

*Glycyrrhiza uralensis* Fisch., as an important windbreak and sand fixation plant in desert areas, widely grown in arid and semiarid regions of the world, especially in the harsh northwestern region of China, and suffers from salt stress year-round (Li et al., 2016; Wang C. et al., 2020). *Bacillus cereus* (G2), as an endophyte found by our laboratory, enhanced several carbohydrate contents in *G. uralensis* under salt stress, but the mechanism has not been thoroughly studied (Zhang, unpublished). During their seedling stage, plants are sensitive to adverse external factors; therefore, the seedlings stage is the optimum time to research plants’ abiotic tolerance (El Atta et al., 2016). Therefore, in order to reveal the behind mechanism of G2 on carbohydrate metabolism in *G. uralensis* seedlings subjected to salt stress, this study analyzed parameters related to photosynthesis, carbohydrate transformation, and EMP pathway-TCA cycle at physiological biochemistry and transcriptome levels. This study not only provided valuable information for exploring the mechanism of G2 improving the salt tolerance of *G. uralensis*, but also laid the foundation for cultivating high-quality and high-yield *G. uralensis*.

## Materials and Methods

### Plant Material

*Glycyrrhiza uralensis* seeds were collected from wild *G. uralensis* plants in Urad front flag, Inner Mongolia, China, in September, 2019. Healthy seeds were selected and stored in a kraft paper bag at 4°C until use.

### *Bacillus cereus* G2 Material and Culture Suspension

*Bacillus cereus* (G2) was isolated from *G. uralensis* roots and identified by Sangon Biotech (Shanghai) Co., Ltd. This strain was deposited at the China General Microbiological Culture Collection Center under the accession No. CGMCC No. 16671, and under the accession No. MT803148 in NCBI.

The strain of G2 was cultured at 30°C for 2 days in a beef extract peptone AGAR medium, which contained 1-g L^–1^ tryptone, 3-g L^–1^ beef extract, 5-g L^–1^ NaCl, and 16-g L^–1^ agar, and the pH of the medium after autoclaving was 6.9–7.1. Then, G2 was grown in a sterilized LB liquid medium, which contained 2.5-g tryptone, 0.75-g beef extract, 1.25-g NaCl, and 250-ml distilled water, and the pH of the medium after autoclaving was 6.9–7.1. The bacteria culture suspension was incubated in a shaking incubator of 180 rpm at 28°C for 2 days.

### Plant Growing Condition and Treatment

All of the seeds of *G. uralensis* were steeped with concentrated sulfuric acid (H_2_SO_4_) for 1.5 h, and then surface sterilized with 0.1% (v/v) H_2_O_2_ for 10 min, rinsed for three times in distilled water, and soaked in distilled water for 9 h at room temperature. Then, 75 water-absorbing, full-filled seeds were selected, blotted surface moisture, and sown evenly in each pot filled with 1,900-g high-pressure sterilized and a fully dried sand medium collected from native desert regions, which did not contain any nutrients. Then, pre-irrigated with 300 ml of distilled water as the control group (CK) or with 300 ml of distilled water containing 75-mM NaCl as the salt stress group (S). The above experiment was carried out in an indoor environment with natural light and an air temperature of 23–28°C during the day and night. After 35 days, G2 treatment was initiated when the third true leaf appeared in most of the *G. uralensis* seedling. The bacteria culture suspension with G2 was centrifuged at 8,000 rpm for 10 min, and it was washed in sterile distilled water, and the optical density (600 nm) of the bacterial strain was adjusted to 1 (∼10^8^ cfu ml^–1^) using sterile distilled water. Both CK and S groups were divided into two subgroups, and were watered either 300-ml distilled water or 300-ml of distilled water, containing 10^8^-cfu ml^–1^ G2. Three replications per treatment were used, and each replication comprised two pots. All pots were randomly arranged and periodically rotated to minimize the effects of environmental heterogeneity.

At 10 days after G2 treatment, the plants were collected and used in subsequent experiments. Generally, three pots of each treatment, firstly, the numbers of plants per pot were recorded for calculation of the survival rate, and all the plants in the pot were taken out of the soil, and then growth indicators were recorded; then, the samples were oven dried at 60°C for 48 h and weighed. The rest three pots of each treatment – all seedlings were taken out of the soil between 9:00 a.m. and 10:00 a.m. and cleaned immediately with distilled water, and then parts of leaves were collected and mixed for determination of Chl contents, and the rest samples were immediately stored at −80°C to measure their physiological and biochemical characteristics.

### Measurement of Photosynthetic Pigment Contents

About 0.2-g fresh leaves material of *G. uralensis* was extracted with 15 ml of a solution that contained ethanol and acetone (1:2, v/v) for 24 h at room temperature in the dark. To calculate the contents of Chl *a*, Chl *b*, and carotenoids (Car), the absorbance was measured at 663, 645, and 470 nm, respectively (Wellburn, 1994).

### Measurement of Carbohydrate Contents

About 0.2-g fresh *G. uralensis* seedlings material was homogenized in 2-ml 80% (v/v) ethanol, and then 80°C water bath for 30 min and centrifuged at 1,000 rpm for 5 min, and the supernatants were analyzed for total soluble sugar (TSS), sucrose, fructose, and starch contents. The TSS content was analyzed by anthrone colorimetry (Li et al., 2020), specifically 100-μL supernatant was mixed with 5-ml anthracite reagent, and incubation was performed for 10 min at 100°C, 630-nm colorimetric analysis. The contents of sucrose and fructose were analyzed by Tang (1999), as follows: Sucrose analysis: 400-μL supernatant added to 200-μL 2-M NaOH that was boiled for 5 min, and then added 3-ml 30% (v/v) HCl, 1-mL.1% m-dihydroxybenzene, which were 80°C water bathed for 10 min, 480-nm colorimetric analysis. The mixtures for the fructose analysis contained 100-μL supernatant, 0.2-ml 80% (v/v) ethanol, 0.8-ml 0.1% m-dihy-droxybenzene, and 2.8-ml 30% (v/v) HCl, and then were 80°C water bathed for 10 min, 480-nm colorimetric analysis. The residues were extracted with 2 ml of 9.2-M perchloric acid, the volumes were adjusted to 10 ml in volumetric flasks, and the supernatants were analyzed to determine the starch contents by anthrone colorimetry (Gao, 2006).

### Measurement of Carbohydrate Transformation-Related Enzyme Activities

About 0.2-g fresh *G. uralensis* seedlings material was homogenized in 2-ml water and centrifuged at 3,000 rpm for 10 min. Then, the amylase determination was carried out according to the method of Devi et al. (2007) with minor modification. α-Amylase (AM) activity was determined after destroying the β-amylase (BM) by heating the enzyme at 70°C for 20 min and estimating reducing sugars formed from 2% starch in a 50-mM sodium acetate buffer (pH 5.0) in the presence of 1-mM CaCl_2_ at 37°C. BM was extracted with a 100-mM sodium acetate buffer (pH 3.6), containing 1-mM EDTA. The activity of BM was determined by estimating the reducing sugars formed after the enzyme action on 1% starch prepared in a 50-mM sodium acetate buffer (pH 5.0), containing 1-mM EDTA. The soluble starch synthase (SSS), granule-bound starch synthase (GBSS), and ADP-glucose pyrophosphorylase (AGP) were determined using reagent kits (Beijing Solarbio Science & Technology Co., Ltd.) according to the manufacturer’s instructions. The GBSS, SSS, and AGP were determined by recording the increase of NADPH at 340 nm. The sucrose phosphate synthase (SPS) and sucrose synthase (SS) activities were measured using the m-dihydroxybenzene method (Tang, 1999) with minor modification. The mixtures for the SPS analysis contained 50-mM Tris–HCl (pH 7.0), 10-mM MgCl_2_, 10-mM fructose-6-phosphate, 3-mM UDP-glucose, and 100 μL of crude enzyme. For the SS activity, the reaction mixtures contained 50-mM Tris–HCl (pH 7.0), 10-mM MgCl_2_, 10-mM fructose, 3-mM UDP-glucose, and 100 μL of crude enzyme. The invertase extraction and determination were carried out according to the method of Scholes et al. (1996). About 0.2-g fresh *G. uralensis* seedlings material was homogenized on an ice bath in 3 ml of extracting solution and centrifuged at 10,000 rpm for 10 min, and enzyme activity was detected in the supernatants. The amount of the compound formed by the reaction of reducing sugar with 3,5-dini-trosalicylic acid indicates its activity, and the absorbance is measured at 510 nm.

### Measurement of Intermediate Contents in the EMP and Tricarboxylic Acid Cycle Pathway

The pyruvate (PA) and citrate (CA) contents were determined using reagent kits (Beijing Solarbio Science & Technology Co., Ltd.) according to the manufacturer’s instructions. PA content was determined using 2,4-dinitrophenylhydrazine as the substrate to record the absorbance at 520 nm. CA content was determined by the reduction of Cr^6+^ at 545 nm.

### Measurement of Enzyme Activities in the EMP and Tricarboxylic Acid Cycle Pathway

The hexokinase (HK), phosphofructokinase (PFK), and pyruvate kinase (PK) were determined using reagent kits (Beijing Solarbio Science & Technology Co., Ltd.) according to the manufacturer’s instructions. HK was determined using glucose as the substrate to record the increase in absorbance at 340 nm caused by the enzymatic reduction of NADP^+^. PFK was determined using 6-phosphate-fructose as the substrate to record the decrease in absorbance at 340 nm caused by the enzymatic oxidation of NADH. PK was determined using phosphoenolpyruvate (PEP) as the substrate to record the decrease in absorbance at 340 nm caused by the enzymatic oxidation of NADH.

The malate dehydrogenase (MDH), pyruvate dehydrogenase (PDH), isocitrate dehydrogenase (ICDH), and succinate dehydrogenase (SDH) were determined using reagent kits (Beijing Solarbio Science & Technology Co., Ltd.) according to the manufacturer‘s instructions. The MDH activity was determined using oxaloacetate as the substrate to record the decrease in absorbance at 340 nm caused by the enzymatic oxidation of NADH. PDH catalyzed dehydrogenation of PA and reduced 2,6-DCPIP at the same time. The PDH activity was determined by recording a decrease in absorbance at 605 nm. The ICDH activity was determined by measuring ketoglutaric acid production at 340 nm, and the SDH activity was determined by recording the reduction rate of 2,6-DCPIP at 600 nm.

### RNA Extraction, cDNA Library Construction, and RNA-Seq

Transcriptome sequencing was carried out by Beijing Baimeike Company. The experimental process followed the method provided by Oxford Nanopore Technologies (ONT), which mainly included the following steps: (i) *G. uralensis* seedlings collected of four treatments (CK, CK + G2, S, and S + G2) were grounded, grouped by weight, and biologically replicated for RNA preparation. Three biological replicates per sample were used for the RNA-Seq experiments. (ii) Total RNA was extracted from the tissue using TRIzol reagent (Takara, Kyoto, Japan) according to the manufacturer’s instructions. RNA purity was tested using the Nano Photometer spectrophotometer (IMPLEN, Westlake Village, United States). (iii) Library construction: primer annealing, reverse transcription into cDNA, plus switch Oligo; synthesis of complementary chains; DNA damage repair and terminal repair and magnetic bead purification. (iv) The cDNA libraries were sequenced using the Illumina NovaSeq 6000 platform.

### Transcriptome Data Assembly

Filter the low-quality (length less than 500 bp, Qscore less than 7) sequence and ribosomal RNA sequence from the original landing sequence, and obtain the full-length sequence according to the presence of primers at both ends of the sequence. Polish the full-length sequence obtained in the previous step to obtain the consistent sequence. Contig comparisons with reference genomes or constructed contig sequences were performed to remove redundancy.

### Transcription Quantification

Transcriptome sequencing can be simulated as a random sampling process. In order to make the number of fragments truly reflect the expression level of transcripts, it is necessary to normalize the number of mapped reads in the sample. CPM (counts per million) (Zhou et al., 2014) was used as an indicator to measure the expression level of transcripts or genes. The calculation formula of CPM was as follows (reads mapped to a transcript means the number of reads compared to the transcript; total reads aligned in the sample represent the total number of fragments compared to the reference transcriptome):


$$\text{CPM}=\frac{\text{reads}mappedtotranscript}{totalreadsalignedinsample}\times 1,000,000$$

### Quantification of Gene/Transcript Expression Levels and Differential Expression Analysis

Full length reads were mapped to the reference transcriptome sequence. Reads with match quality above 5 were further used to quantify. Expression levels were estimated by reads per gene/transcript per 10,000 reads mapped. For the samples with biological replicates: differential expression analysis of two conditions/groups was performed using the DESeq R package (1.18.0). DESeq provided statistical routines for determining differential expression in digital gene expression data using a model based on the negative binomial distribution. The resulting *p*-values were adjusted using the Benjamini and Hochberg’s approach for controlling the false discovery rate. Genes with a *p*-value < 0.05 and fold change ≥ 1.5 found by DESeq were assigned as differentially expressed.

### Reverse Transcriptase-Polymerase Chain Reaction and Quantitative Real-Time-Polymerase Chain Reaction

Reverse transcriptase-polymerase chain reaction RT-PCR and quantitative real-time-polymerase chain reaction (qRT-PCR) were performed on the same RNA pools, which were previously used in RNA-Seq for 10 key DEGs of carbohydrate metabolism in*G. uralensis*, respectively. Gene-specific primers were designed based on the sequencing data using the Primer-BLAST tool. The primer sequences used for conventional PCR and qRT-PCR analysis are listed in Supplementary Table 1. First-strand cDNAs were synthesized from 2 μg of DNase-treated total RNA using RevertAid RT Reverse Transcription Kit (Servicebio, China). As for RT-PCR, target genes were amplified using a polymerase chain reaction (PCR) thermal cycler (ABI 2720, United States), photographed by FR-980B gel imaging system (FuRi Science & Technology Co., Ltd.), and analyzed with pixel quantitation software Image J 2 (Rawak Software Inc.). qRT-PCR was performed by Wuhan Servicebio Technology CO., LTD. using 2 × SYBR Green qPCR Master Mix (High ROX). The reaction mixture (20 μL) contained 2 × ChamQ SYBR Green qPCR Master Mix 10 μL, 1 μL of primer, 1 μL of template cDNA,.4 μL of 50 × ROX Reference Dye 1 and 7.6 μL of ddH2O. Amplifications were performed under the following conditions: 95°C for 10 min, followed by 40 cycles of 95°*C* for 15 s and 60°*C* for 60 s. Relative expression for each sample was calculated using the 2^–ΔΔCt^ methods with normalization to the internal control genes.

### Statistical Analyses

All treatments have three replication operations presented as the mean ± SD of each experiment. The one-way ANOVA was carried out, and physiological data were tested for significant treatment differences using Duncan’s multiple range tests, and *p* < 0.05 was considered to be statistically significant. The correlation analysis of photosynthetic pigments contents, carbohydrates contents, enzymes activities, and intermediates contents of carbohydrate transformation and EMP pathway-TCA cycle in *G. uralensis* under four different conditions was determined. Principal component analysis (PCA) of the response variables photosynthetic pigments contents, carbohydrates contents, enzymes activities, and intermediates contents of carbohydrate transformation and EMP pathway-TCA cycle was performed to separate plants in different treatments. The first two principal components, which accounted for the highest variation, were then used to plot two-dimensional scatter plots. The qRT-PCR data were tested for significant treatment differences using the Student’s *T*-test, and *p* < 0.05 was considered to be statistically significant. All statistical analyses were performed using SPSS Statistics 25, Origin 2018 Statistics, and GraphPad Prism 8.0.1.

## Results

Effect of *Bacillus cereus* G2 on the Growth Parameters of *Glycyrrhiza uralensis* Seedlings

**FIGURE 1:**
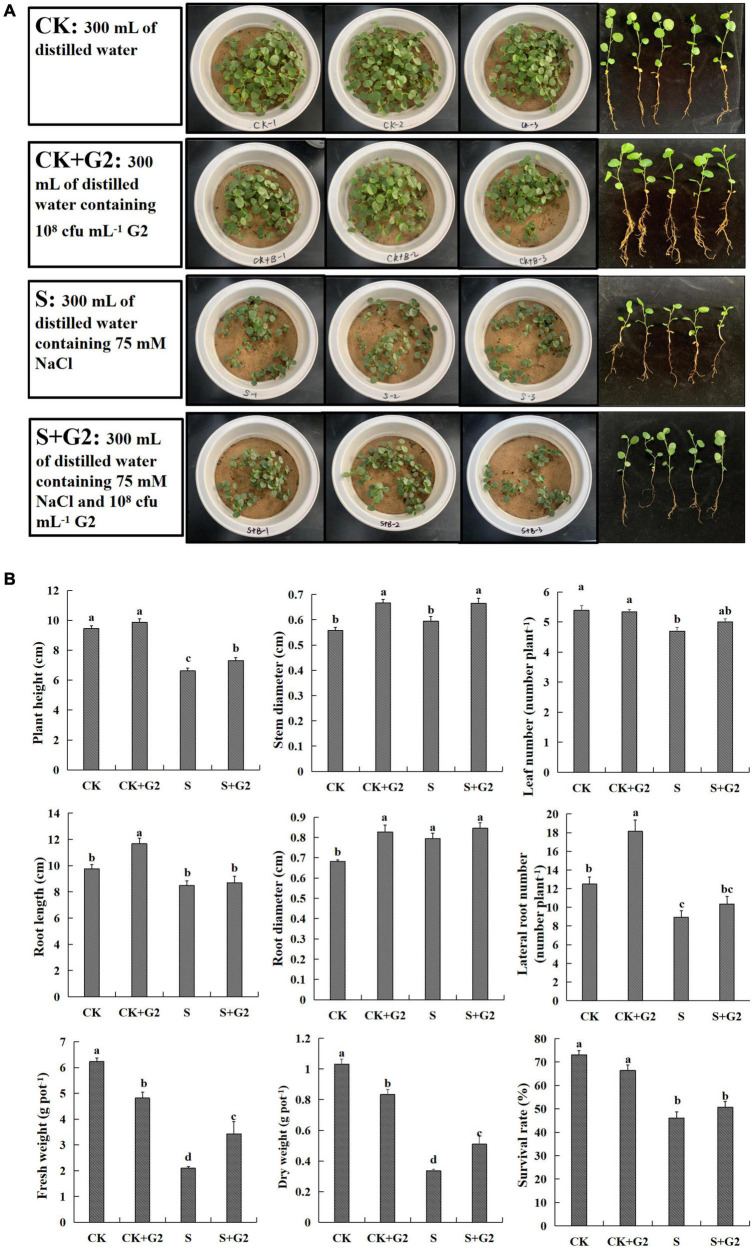
**(A)** A part of the experiment layout of *Glycyrrhiza uralensis* Fisch. seedlings grown in control and salt stress conditions without or with *Bacillus cereus* G2. **(B)** Effects of *B. cereus* G2 on growth parameters of *G. uralensis* seedlings under control and salt stress conditions. CK, control group; S, salt stress group; CK + G2, control combined with the G2 group; S + G2, salt stress combined with the G2 group. The different letters within the different treatments in the same parameter indicate the significant difference at the 0.05 level. Values are means ± SE (*n* = 3).

The growth situation of *G. uralensis* seedling under all treatments (CK, CK + G2, S, and S + G2) is shown in Figure 1A. Salt stress significantly decreased plant height, leaf number, lateral root number, fresh weight, dry weight, and survival rate, but increased the root diameter. Interestingly, G2 significantly increased plant height, stem diameter, fresh weight, and dry weight of *G. uralensis* under salt stress (Figure 1B).

### Effect of *Bacillus cereus* G2 on Carbohydrate Contents in *Glycyrrhiza uralensis* Seedlings

**FIGURE 2:**
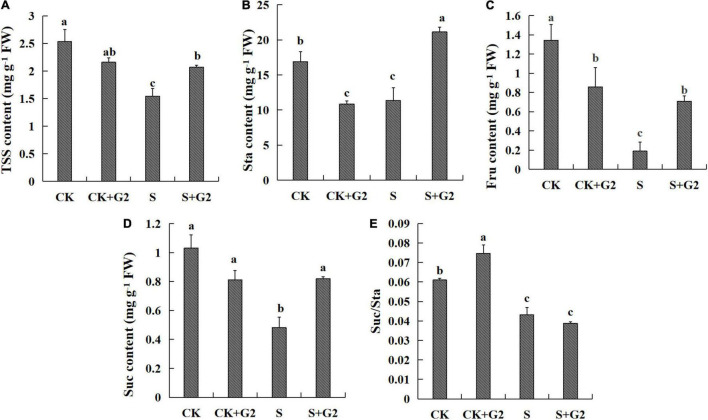
Effects of *B. cereus* G2 on carbohydrates contents in *G. uralensis* seedlings under control and salt stress conditions. CK, control group; S, salt stress group; CK + G2, control combined with the G2 group; S + G2, salt stress combined with the G2 group. The different letters within the different treatments in the same parameter indicate the significant difference at the 0.05 level. Values are means ± SE (*n* = 3). **(A)** TSS: total soluble sugar. **(B)** Sta: starch. **(C)** Fru: fructose. **(D)** Suc: sucrose. **(E)** Suc/Sta: sucrose/starch.

Salt stress significantly decreased the contents of TSS, starch, fructose, sucrose, and the ratio of sucrose/starch, while G2 significantly increased the contents of TSS, starch, fructose, and sucrose in *G. uralensis* under salt stress (Figure 2). TSS, sucrose, and fructose were very positively correlated with one another (Figure 9 and Supplementary Figure 1A).

### Effect of *Bacillus cereus* G2 on Photosynthesis in *Glycyrrhiza uralensis* Seedlings

**FIGURE 3:**
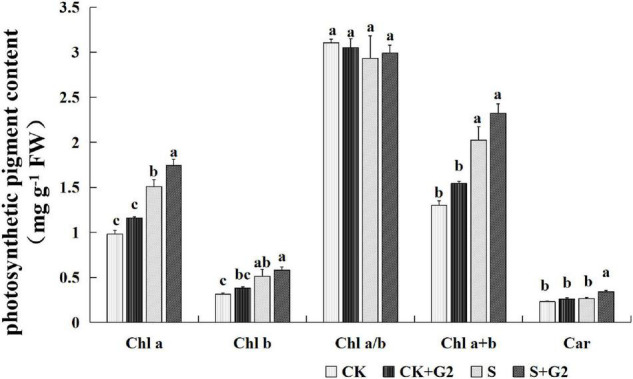
Effects of *B. cereus* G2 inoculation on photosynthetic pigment contents in *G. uralensis* seedlings under control and salt stress conditions. CK, control group; S, salt stress group; CK + G2, control combined with the G2 group; S + G2, salt stress combined with the G2 group. The different letters within the different treatments in the same parameter indicate the significant difference at the 0.05 level. Values are means ± SE (*n* = 3). Chl *a*, chlorophyll *a*; Chl *b*, chlorophyll *b*; Car, carotenoids.

Salt stress significantly increased Chl *a*, Chl *b*, and Chl *a* + *b* contents in *G. uralensis*. Interestingly, G2 significantly further increased the Chl *a* and Car contents in *G. uralensis* under salt stress (Figure 3).

**FIGURE 4:**
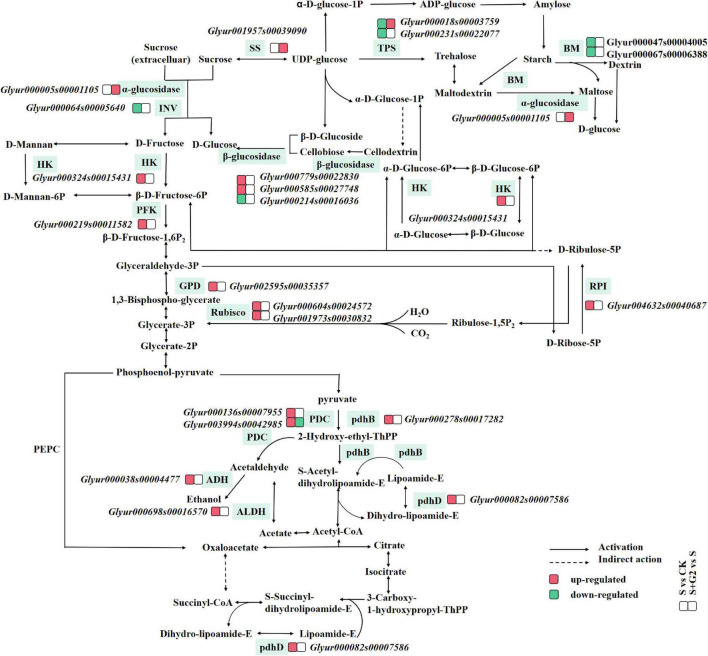
A figure of the mechanism by which *B. cereus* G2 improves the salt tolerance of *G. uralensis* by regulating DEGs related to carbohydrate metabolism. CK, control group; S, salt stress group; S + G2, salt stress combined with the G2 group; GPD, glyceraldehyde-3-phosphate dehydrogenase (NADP+); Rubisco, ribulose-bisphosphate carboxylase small chain; RPI, ribose 5-phosphate isomerase A; INV, beta-fructofuranosidase; TPS, trehalose 6-phosphate synthase/phosphatase; BM, beta-amylase; SS, sucrose synthase; HK, hexokinase; PFK, 6-phosphofructokinase 1; pdhB, pyruvate dehydrogenase E1 component beta subunit; PDC, pyruvate decarboxylase; ADH, S-(hydroxymethyl)glutathione dehydrogenase/alcohol dehydrogenase; pdhD, dihydrolipoamide dehydrogenase; ALDH, aldehyde dehydrogenase.

In carbon fixation in the photosynthetic organisms pathway (Table 1 and Figure 4) according to transcriptomic analysis, glyceraldehyde-3-phosphate dehydrogenase (NADP^+^) (GPD), ribulose-bisphosphate carboxylase small chain (Rubisco), and ribose 5-phosphate isomerase (RPI) A were differentially expressed in S vs. CK comparison. Specifically, the gene (*Glyur002595s00035357*)-encoding GPD, the genes (*Glyur000604s00024572* and *Glyur001973s00030832*)-encoding Rubisco, and the gene (*Glyur004632s00040687*)-encoding RPI were upregulated in S vs. CK comparison. However, G2 had no significant effect on the genes related to carbon fixation in the photosynthetic organisms pathway.

**TABLE 1:** Regulation of carbohydrate metabolism-related DEGs in *G. uralensis* by salt stress or *B. cereus* G2.

Pathway	Enzyme name	Definition (EC)	Gene ID	Regulated	
				S vs. CK comparison	S + G2 vs. S comparison
Carbon fixation in photosynthetic organisms	GPD	Glyceraldehyde-3-phosphate dehydrogenase (NADP+) (phosphorylating) (EC:1.2.1.13)	*Glyur002595s00035357*	Up	–
	Rubisco	Ribulose-bisphosphate carboxylase small chain (EC:4.1.1.39)	*Glyur000604s00024572*	Up	–
			*Glyur001973s00030832*	Up	–
	RPI	Ribose 5-phosphate isomerase (EC:5.3.1.6)	*Glyur004632s00040687*	Up	–
Starch and sucrose metabolism	β-Glucosidase	Beta-glucosidase (EC:3.2.1.21)	*Glyur000779s00022830*	Up	–
			*Glyur000585s00027748*	Up	–
			*Glyur000214s00016036*	Down	–
	INV, sacA	Beta-fructofuranosidase (EC:3.2.1.26)	*Glyur000064s00005640*	Down	–
	α-Glucosidase	Alpha-glucosidase (EC:3.2.1.20)	*Glyur000005s00001105*	–	Up
	TPS	Trehalose 6-phosphate synthase/phosphatase (EC:2.4.1.15 3.1.3.12)	*Glyur000018s00003759*	Down	Up
			*Glyur000231s00022077*	Down	–
	BM	Beta-amylase (EC:3.2.1.2)	*Glyur000047s00004005*	Down	–
			*Glyur000067s00006388*	Down	–
	SS	Sucrose synthase (EC:2.4.1.13)	*Glyur001957s00039090*	–	Up
	HK	Hexokinase (EC:2.7.1.1)	*Glyur000324s00015431*	Up	–
EMP-TCA cycle	HK	Hexokinase (EC:2.7.1.1)	*Glyur000324s00015431*	Up	–
	PFK, pfkA	6-Phosphofructokinase 1 (EC:2.7.1.11)	*Glyur000219s00011582*	Up	–
	PDC	Pyruvate decarboxylase (EC:4.1.1.1)	*Glyur000136s00007955*	Up	–
			*Glyur003994s00042985*	Up	Down
	ADH	S-(hydroxymethyl)glutathione dehydrogenase/alcohol dehydrogenase (EC:1.1.1.284 1.1.1.1)	*Glyur000038s00004477*	Up	–
	ALDH	Aldehyde dehydrogenase (NAD+) (EC:1.2.1.3)	*Glyur000698s00016570*	Up	–
	pdhB, PDHB	Pyruvate dehydrogenase E1 component beta subunit (EC:1.2.4.1)	*Glyur000278s00017282*	Up	–
	pdhD, DLD, lpd	Dihydrolipoamide dehydrogenase (EC:1.8.1.4)	*Glyur000082s00007586*	Up	–

*CK, control group; S, salt stress group; S + G2, salt stress combined with G2 group.*

*“–” means normal.*

In the relationship between carbohydrates and photosynthetic pigments (Figure 9 and Supplementary Figure 1A), fructose content was negatively correlated with Chl *a* and Chl *a* + *b* contents; the ratio of sucrose/starch was very negatively correlated with Chl *a* and Chl *a* + *b* contents, and negatively correlated with Chl *b* and Car contents.

### Effect of *Bacillus cereus* G2 on Enzymes Related to Carbohydrate Transformation in *Glycyrrhiza uralensis* Seedlings

**FIGURE 5:**
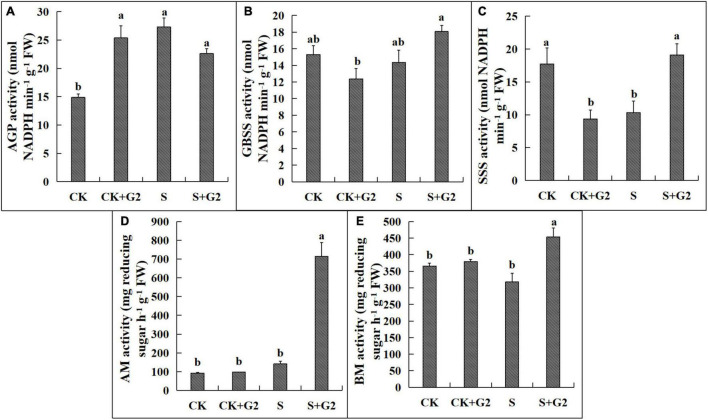
Effects of *B. cereus* G2 on enzymes related to starch synthesis and decomposition in *G. uralensis* seedlings under control and salt stress conditions. CK, control group; S, salt stress group; CK + G2, control combined with the G2 group; S + G2, salt stress combined with the G2 group. The different letters within the different treatments in the same parameter indicate the significant difference at the 0.05 level. Values are means ± SE (*n* = 3). **(A)** AGP: ADP-glucose pyrophosphorylase. **(B)** GBSS: granule-bound starch synthase. **(C)** SSS: soluble starch synthase. **(D)** AM: α-amylase; **(E)** BM: β-amylase.

**FIGURE 6:**
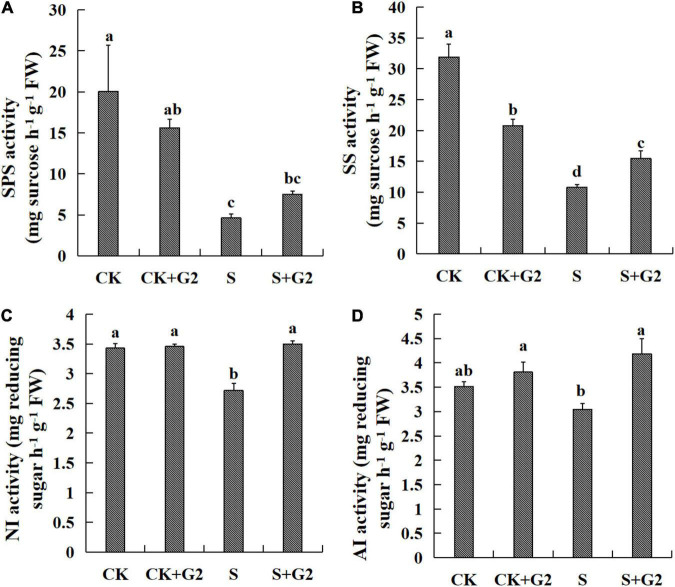
Effects of *B. cereus* G2 on enzymes related to sucrose synthesis and decomposition in *G. uralensis* seedlings under control and salt stress conditions. CK, control group; S, salt stress group; CK + G2, control combined with the G2 group; S + G2, salt stress combined with the G2 group. The different letters within the different treatments in the same parameter indicate the significant difference at the 0.05 level. Values are means ± SE (*n* = 3). **(A)** SPS: sucrose-phosphate synthase. **(B)** SS: sucrose synthase. **(C)** NI: neutral invertase. **(D)** AI: acid invertase.

Salt stress significantly increased AGP activity but decreased SSS activity, while G2 significantly increased the activities of SSS, AM, and BM in *G. uralensis* under salt stress (Figures 5, 8). Salt stress significantly decreased the activities of SPS, SS, and NI, while G2 significantly increased the activities of SS, NI, and AI in *G. uralensis* under salt stress (Figures 6, 8).

In the starch and sucrose metabolism pathway (Table 1 and Figure 4), β-glucosidase, β-fructofuranosidase (INV), α-glucosidase, trehalose 6-phosphate synthase/phosphatase (TPS), BM, SS, and HK were differentially expressed under different treatments. Specifically, the genes (*Glyur000779s00022830* and *Glyur000585s00027748*)-encoding β-glucosidase and the gene (*Glyur000324s00015431*)-encoding HK were upregulated, but the gene (*Glyur000214s00016036*)-encoding β-glucosidase, the gene (*Glyur000064s00005640*)-encoding INV, the genes (*Glyur000018s00003759* and *Glyur000231s00022077*)-encoding TPS and the genes (*Glyur000047s00004005* and *Glyur000067s00006388*)-encoding BM were downregulated in S vs. CK comparison. However, the gene (*Glyur000005s00001105*)-encoding α-glucosidase, the gene (*Glyur000018s00003759*)-encoding TPS, and the gene (*Glyur001957s00039090*)-encoding SS were upregulated in S + G2 vs. S comparison.

In the relationship between carbohydrate and carbohydrate transformation parameters (Figure 9 and Supplementary Figure 1B), TSS content was very positively correlated with SS and SPS activities and positively correlated with NI activity, and very negatively correlated with AGP activity. Starch content was very positively correlated with SSS and AM and positively correlated with the activities of GBSS and BM. Sucrose and fructose contents were very positively correlated with SS activity and positively correlated with NI activity, and very negatively correlated with AGP activity. The ratio of sucrose/starch was positively correlated with SPS activity and negatively correlated with the activities of GBSS and AM.

### Effect of *Bacillus cereus* G2 on the Levels of Intermediates and Enzymes in the EMP Pathway-Tricarboxylic Acid Cycle of *Glycyrrhiza uralensis* Seedlings

**FIGURE 7:**
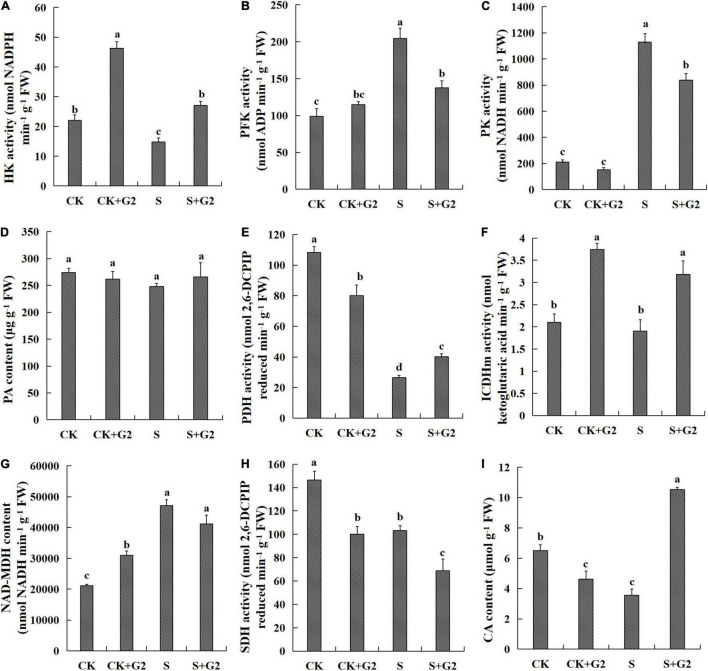
Effects of *B. cereus* G2 on EMP-TCA-related enzyme activities and intermediate contents in *G. uralensis* seedlings under control and salt stress conditions. CK, control group; S, salt stress group; CK + G2, control combined with the G2 group; S + G2, salt stress combined with the G2 group. The different letters within the different treatments in the same parameter indicate the significant difference at the 0.05 level. Values are means ± SE (*n* = 3). **(A)** HK: hexokinase. **(B)** PFK: phosphofructokinase. **(C)** PK: pyruvate kinase. **(D)** PA: pyruvate. **(E)** PDH: pyruvate dehydrogenase. **(F)** ICDH: isocitrate dehydrogenase. **(G)** MDH: malate dehydrogenase. **(H)** SDH: succinate dehydrogenase. **(I)** CA: citrate.

**FIGURE 8:**
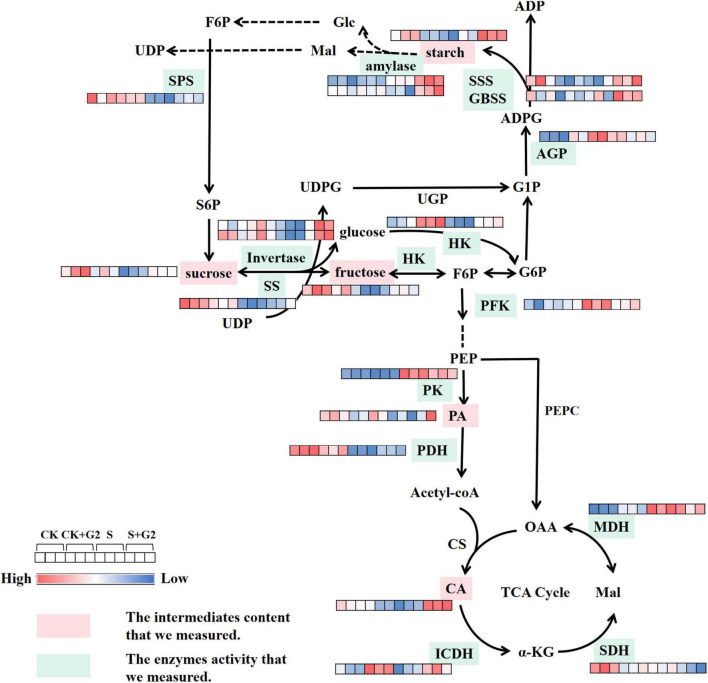
A figure of the mechanism by which *B. cereus* G2 improves the salt tolerance of *G. uralensis* by regulating enzymes and intermediates related to carbohydrate metabolism. CK, control group; S, salt stress group; CK + G2, control combined with the G2 group; S + G2, salt stress combined with the G2 group. SPS, sucrose-phosphate synthase; SS, sucrose synthase; AGP, ADP-glucose pyrophosphorylase; GBSS, granule-bound starch synthase; HK, hexokinase; PFK, phosphofructokinase; PK, pyruvate kinase; PA, pyruvate; PDH, pyruvate dehydrogenase; CA, citrate; ICDH, isocitrate dehydrogenase; SDH, succinate dehydrogenase; MDH, malate dehydrogenase.

In the EMP pathway, salt stress significantly decreased HK activity, while increased PFK and PK activities in *G. uralensis*. Interestingly, G2 only significantly increased HK activity under salt stress but decreased PK and PFK activities in *G. uralensis* under salt stress. As for the TCA cycle, salt stress significantly decreased PDH and SDH activities and CA content, but increased MDH activity in *G. uralensis*. Interestingly, G2 significantly increased PDH and ICDH activities and CA content, but decreased SDH activity under salt stress condition (Figures 7, 8).

**FIGURE 9:**
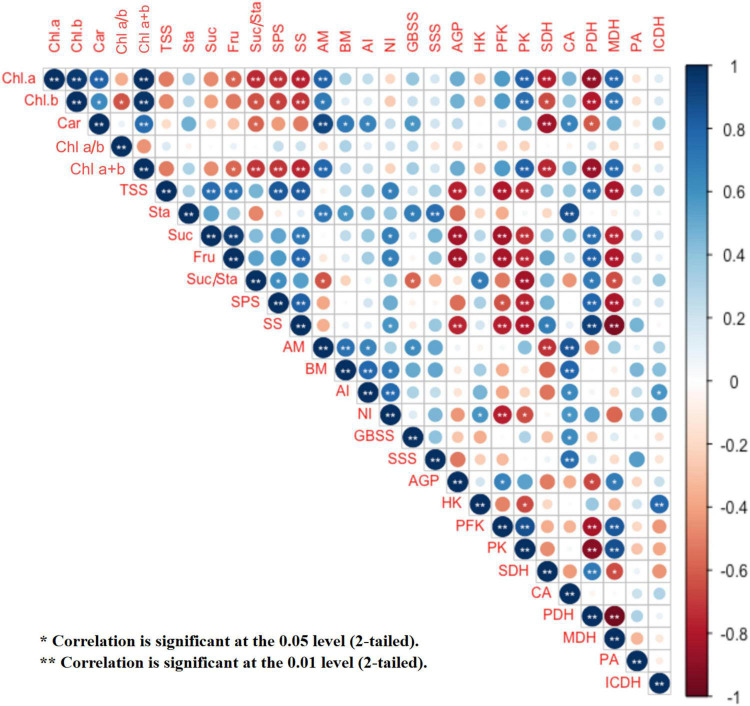
Correlation analysis among *G. uralensis* photosynthetic pigment contents, starch and sucrose metabolism, EMP and TCA cycle indexes in the four treatments (CK, CK + G2, S, and S + G2). CK, control group; S, salt stress group; CKEE + G2, control combined with the G2 group; S + G2, salt stress combined with the G2 group. Chl *a*, chlorophyll *a*; Chl *b*, chlorophyll *b*; Car, carotenoids; AGP, ADP-glucose pyrophosphorylase; GBSS, granule-bound starch synthase; SSS, soluble starch synthase; AM, α-amylase; BM, β-amylase; SPS, sucrose-phosphate synthase; SS, sucrose synthase; AI, acid invertase; NI, neutral invertase; HK, hexokinase; PFK, phosphofructokinase; PK, pyruvate kinase; PDH, pyruvate dehydrogenase; ICDH, isocitrate dehydrogenase; SDH, succinate dehydrogenase; MDH, malate dehydrogenase; PA, pyruvate; CA, citrate; TSS, total soluble sugar; Sta, starch; Fru, fructose; Suc, sucrose.

In the EMP pathway-TCA cycle (Table 1 and Figure 4), HK, PFK, pyruvate decarboxylase (PDC), S-(hydroxymethyl)glutathione dehydrogenase/alcohol dehydrogenase (ADH), aldehyde dehydrogenase (NAD^+^) (ALDH), pyruvate dehydrogenase E1 component beta subunit (pdhB), and dihydrolipoamide dehydrogenase (pdhD) were differentially expressed under different treatments. Specifically, the gene (*Glyur000324s00015431*)-encoding HK, the gene (*Glyur000219s00011582*)-encoding PFK, the genes (*Glyur000136s00007955* and *Glyur003994s00042985*)-encoding PDC, the gene (*Glyur000038s00004477*)-encoding ADH, the gene (*Glyur000698s00016570*)-encoding ALDH, the gene (*Glyur000278s00017282*)-encoding pdhB, and the gene (*Glyur000082s00007586*)-encoding pdhD were upregulated in S vs. CK comparison, while only the gene (*Glyur003994s00042985*)-encoding PDC was downregulated in S + G2 vs. S comparison.

In the relationship between EMP pathway-TCA cycle parameters and carbohydrates (Figure 9 and Supplementary Figure 1C), the HK activity was positively correlated with the ratio of sucrose/starch; the PFK activity was very negatively correlated with the contents of TSS, sucrose, and fructose; the PK activity was very negatively correlated with the contents of TSS, sucrose, fructose, and the ratio of sucrose/starch; the PDH activity was very positively correlated with the contents of TSS, sucrose, and fructose and positively correlated with the ratio of sucrose/starch; the MDH activity was very negatively correlated with the contents of TSS, sucrose, and fructose and negatively correlated with the ratio of sucrose/starch; the CA content was only very positively correlated with the starch content.

### Principal Component Analysis

The results of PCA related to photosynthetic pigment contents, starch, and sucrose metabolism and EMP pathway-TCA cycle indexes that we measured revealed a closer association between biological replicates than between salinity or G2 treatments (Supplementary Figures 2A,B). PC1 explaining 42.9% of total variation uncovered differences between plants of CK and S treatments, while PC2, accounting for 26.5% of total variation, distinctly separated the plants provided with S and S + G2 treatments. PC1 formation covers photosynthetic pigment contents, sucrose synthesis, and EMP indices, while PC2 formation covers starch synthesis and decomposition indices. PC1 clearly separated S-treated *G. uralensis* from control *G. uralensis* because of photosynthetic pigment contents, sucrose synthesis, and EMP indices. PC2 clearly separated S + G2-treated *G. uralensis* from S-treated *G. uralensis* because of starch synthesis and decomposition indices.

The results of PCA related to gene expression in carbon fixation in photosynthetic organisms, starch, and sucrose metabolism and the EMP pathway-TCA cycle pathway also revealed a relatively closer association between biological replicates than between salinity or G2 treatments (Supplementary Figures 2C,D). PC1 explaining 44.2% of total variation revealed differences between plants of CK and S treatments, while PC2 accounting for 19.2% of total variation distinctly separated the plants provided with S and S + G2 treatments, which is consistent with the result of the PCA in the physiological biochemical level. PC1 formation covered the genes related to the synthesis and decomposition of sucrose and fructose, while PC2 formation covers the genes-encoding pdhD, PFK, and PDC-2. PC1 clearly separated S-treated *G. uralensis* from control *G. uralensis* because of the genes related to the synthesis and decomposition of sucrose and fructose indices. PC2 clearly separated S + G2-treated *G. uralensis* from S-treated *G. uralensis* because of the genes-encoding pdhD, PFK, and PDC-2.

### The Polymerase Chain Reaction Expression Level of the Ley DEGs Related to Carbohydrate Metabolism in *Glycyrrhiza uralensis* Seedlings

**FIGURE 10:**
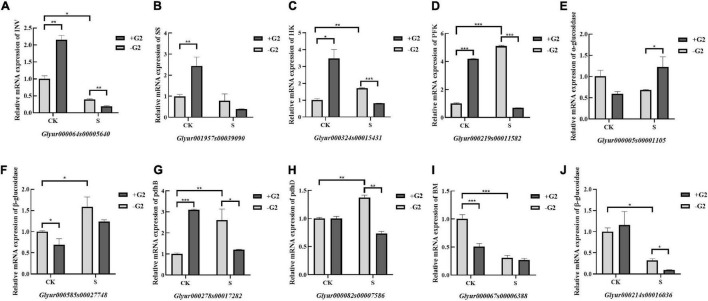
Expression of carbohydrate metabolism-related genes (qRT-PCR) in *G. uralensis* in four treatments (CK, CK + G2, S, and S + G2). CK, control group; S, salt stress group; CK + G2, control combined with the G2 group; S + G2, salt stress combined with the G2 group (mean ± SD; *p < 0.05; **p < 0.01; ***p < 0.001). **(A)** INV: beta-fructofuranosidase. **(B)** SS: sucrose synthase. **(C)** HK: hexokinase. **(D)** PFK: 6-phosphofructokinase 1. **(E)** α-glucosidase: alpha-glucosidase. **(F,J)** β-glucosidase: beta-glucosidase. **(G)** pdhB: pyruvate dehydrogenase E1 component beta subunit. **(H)** pdhD: dihydrolipoamide dehydrogenase **(I)** BM: beta-amylase.

To elucidate the correlation between mRNA transcript levels and gene expression levels and further analyze the carbohydrate metabolism-related genes expression level, transcriptional analysis of 10 DEGs in carbohydrate metabolism was conducted *via* RT-PCR and qRT-PCR in *G. uralensis* seedlings under four treatments (CK, CK + G2, S, and S + G2). The genes-encoding β-glucosidase, INV, α-glucosidase, BM, and SS in carbohydrate transformation and HK, PFK, pdhB, and pdhD in the EMP pathway-TCA cycle were used for this study. The RT-PCR results are shown in Supplementary Figure 3. The qRT-PCR results (Figure 10) showed that salt stress downregulated the genes-encoding INV, BM, and one of the gene-encoding β-glucosidase, while upregulated the genes-encoding HK, PFK, pdhB, pdhD, and one of the gene-encoding β-glucosidase in *G. uralensis* seedlings. G2 downregulated the genes-encoding INV, HK, PFK, pdhB, pdhD, and one of the genes encoding β-glucosidase, while G2 upregulated the genes encoding α-glucosidase under salt stress in *G. uralensis* seedlings.

## Discussion

### Effect of G2 on Growth in *Glycyrrhiza uralensis* Under Salt Stress

Growth inhibition is the most frequent and significant effect of salt stress on plants, and it is mainly manifested as a decrease in biomass (Zhang et al., 2018). Present results also showed that salt stress inhibited the growth of *G. uralensis*, and this effect was partly reversed by G2 inoculation (Figure 2), agreeing with previous findings on wheat (*Triticum turgidum* subsp. *durum*) (Ibarra-Villarreal et al., 2021), soybean (*G. max* L.) (El-Esawi et al., 2018), and *Artemisia ordosica* (Hou et al., 2021). Specifically, G2 significantly increased plant height, stem diameter, fresh weight, and dry weight of *G. uralensis* seedlings under salt stress (Figure 1B).

### Effect of G2 on Photosynthesis in *Glycyrrhiza uralensis* Under Salt Stress

Carbohydrate is produced during photosynthesis and acts as a substrate for respiration, which reflects the balance between plant carbon acquisition and expenditure (Liu et al., 2016). We mainly analyzed the genes in carbon fixation in photosynthetic organisms and the contents of photosynthetic pigments.

Our transcriptomic analysis showed that salt stress upregulated the genes-encoding RPI, Rubisco, and GPD in carbon fixation in photosynthetic organisms. The pathway of carbon fixation in photosynthetic organisms is mainly divided into three stages: the carboxylation stage, the reduction stage, and the regeneration stage. In the carboxylation stage, Rubisco as the key enzyme in the first step converted ribulose-1,5-bisphosphate (RuBP) and atmospheric CO_2_ into two molecules of 3-phosphoglycerate (3-PGA). Salt stress-induced stomatal closure generally causes a decline in the available CO_2_; in this case, Rubisco uses O_2_ that leads to the formation of 2-phosphoglycolate, which is a toxic two-carbon compound and inhibits at least two key enzymes of carbon metabolism, thus attenuates the net photosynthesis rate (Frukh et al., 2019). In our study, salt stress upregulated genes-encoding Rubisco; on the one hand, salt stress promoted the synthesis of 3-PGA, therefore promoted the energy storage process of photosynthesis at the substrate level; on the other hand, salt stress might promote the synthesis of 2-PGA, thereby weakening photosynthesis. However, the direction of Rubisco regulation depends on the partial pressure of CO_2_ and O_2_ in the environment, which need to be further analyzed. In the reduction stage, GPD catalyzes the reduction of 1,3-bisphosphoglycerate by NADPH to produce 3-phosphoglyceraldehyde (G3P) and NADP^+^; then, PGA can accept electrons from NADPH to decrease ROS production, and G3P can be used to synthesize starch in the chloroplast or can be transported to the cytoplasm for sucrose biosynthesis (Chintakovid et al., 2017). In this study, salt stress upregulated the gene-encoding GPD, therefore promoted the energy storage process of photosynthesis. However, whether the upregulation of the GPD gene under salt stress can help *G. uralensis* reduce the production of ROS and promote the synthesis of starch and sucrose remains to be confirmed by further studies. In the regeneration stage, RPI not only isomerizes ribose-5-phosphate into ribulose-5-phosphate but also helps in the regeneration of the Rubisco substrate (Moigne et al., 2020); therefore, the upregulation of the gene-encoding RPI could contribute to providing sufficient Rubisco substrates for *G. uralensis* seedling under salt stress. However, our results found that G2 had little effect on the gene related to carbon fixation in photosynthetic organisms in *G. uralensis* under salt stress.

Salt stress generally reduced Chl content (Rabiei et al., 2020); however, some studies have reported increased Chl content with increasing salt stress in salt-tolerant plants (Borghesi et al., 2011; Huang et al., 2015; Qiu et al., 2017). In our study, salt stress also increased contents of Chl *a*, *b*, and *a* + *b* in *G. uralensis*, and then, maybe, leading to highly efficient photosynthesis in terms of light reaction, which is further supported by our observation that *G. uralensis* leaves under salt stress exhibited greener compared to that under control conditions (Figure 1A). Interestingly, G2 further increased Chl *a* and Car contents in *G. uralensis* exposed to salt stress, which possibly helped improve the photosynthetic rate of *G. uralensis* and promote plants to resist oxidative stress caused by salt stress (Gururani et al., 2015), thus accumulating more photosynthetic products. Carbohydrates are important substrates of metabolism that helps the plants in various physiological events by regulating the import of carbon to the metabolical sink (Aliche et al., 2019). In this study, salt stress resulted in a significant decrease in starch and TSS contents, and decreased *G. uralensis* growth and yield; in this case, *G. uralensis* thus could require the enhanced photosynthetic rate to synthesize more photosynthetic products to maintain the normal metabolism under salt stress, which may be the reason for the increase of Chl content under salt stress. Moreover, G2 significantly increased the starch and TSS contents in *G. uralensis* under salt stress, which could attribute to improve the photosynthetic rate caused by increased Chl *a* and Car contents.

It was concluded that the G2 increased carbohydrate contents mainly by increasing photosynthetic pigments rather than by regulating the gene related to carbon fixation in photosynthetic organisms in *G. uralensis* under salt stress.

### Effect of G2 on Carbohydrate Transformation in *Glycyrrhiza uralensis* Under Salt Stress

Carbohydrate contents are not only related to photosynthesis but also closely related to the key enzymes in its synthesis and decomposition. Maintenance of the balance among the production, translocation, partition, and use of carbohydrate is important for plants’ normal growth. Starch, sucrose, and fructose are the important components of carbohydrate.

Starch is a major storage form of carbohydrate and an energy carrier in plant storage organs. In the present study, salt stress significantly decreased starch content, while G2 significantly increased starch content in *G. uralensis* under salt stress (Figure 2). In starch synthesis, AGP catalyzes the conversion of glucose 1-phosphase and ATP to produce the precursor for starch biosynthesis, ADP-glucose (Sun et al., 2021), while GBSS and SSS catalyze the elongation of a-1,4-glucosidic bonds on amylose and amylopectin molecules, respectively, by the transfer of glucose from ADPG (Wu et al., 2020). Transcriptomic analysis showed that the genes related to starch synthesis had no differential expression in *G. uralensis* both in S vs. CK and S + G2 vs. S comparisons. However, different results were found when the activities of enzymes related to starch synthesis were measured. Specifically, salt stress significantly increased AGP activity but decreased SSS activity, while G2 significantly increased the activity of SSS in *G. uralensis* under salt stress (Figure 3), which could be the result of the combined action of all genes-encoding AGP and SSS. The results indicating that the decrease of starch under salt stress were mainly due to the inhibition of amylopectin synthesis, while the accumulation of starch by G2 under salt stress was also mainly due to the promotion of amylopectin synthesis. In starch decomposition, amylase catalyzes the hydrolysis of starch to a mixture of smaller oligosaccharides (Baslam et al., 2020). Specifically, starch grains are attacked by AM to release linear and branched oligosaccharides, and then BM catalyzes branched oligosaccharides to release maltose disaccharides (Lloyd and Kötting, 2016). G2 had no effect on the genes-encoding AM and BM by transcriptomic analysis and the gene *Glyur000067s00006388* by qRT-PCR, but G2 increased AM and BM activities in *G. uralensis* under salt stress that helped starch hydrolysis and provided energy for subsequent life activities of plants, which could be the result of the combined action of all genes-encoding AM and BM. Furthermore, S + G2 treatment increased AM and BM activities, and the variation activity of AM was higher than BM, which was possibly due to excessive starch content required increased AM activity to decompose and produce linear and branched oligosaccharides, thus providing sufficient substrates for subsequent carbohydrate metabolism, whereas BM only catalyzed branched oligosaccharides. Moreover, starch content was very positively correlated with SSS and AM activities and positively correlated with BM activity (Figure 7), which also indicated that the decrease of starch content in *G. uralensis* under salt stress was mainly attributed to the decrease of SSS activity, while the increase of starch content by G2 in *G. uralensis* under salt stress was also mainly due to the increase of SSS activity firstly, and then the elevated starch required higher activities of amylase for decomposing, which thus improved the activities of AM and BM.

Sucrose is the main product of plant photosynthesis, which not only acts as a carbon and energy source but also plays a key role as a signaling molecule to regulate the growth of source and sink tissues and the sugar-mediated feedback repression of photosynthesis (Gil et al., 2013). In the present study, salt stress markedly decreased sucrose content, while G2 significantly increased sucrose content in *G. uralensis* under salt stress. SPS is responsible for the synthesis of sucrose from glucose and fructose, and AI, NI, and α-glucosidase catalyze the sucrose to glucose and fructose (Lombardo et al., 2011; Bilska-Kos et al., 2020), while SS has a dual role in the synthesis and hydrolysis of sucrose (Yang et al., 2020). In sucrose synthesis, salt stress had no significantly effect on the genes-encoding SPS and SS by transcriptomic analysis and the gene *Glyur001957s00039090* by qRT-PCR, but salt stress significantly decreased SS and SPS activities that inhibited the synthesis of sucrose, which could be the result of the combined action of all genes-encoding SS and SPS. G2 upregulated the gene *Glyur001957s00039090*-encoding SS by transcriptomic analysis, and G2 also increased SS activity in *G. uralensis* under salt stress that promoted the synthesis of sucrose, which suggested that G2 promoted the synthesis of sucrose, mainly by regulating SS activity resulted from upregulation of the gene *Glyur001957s00039090.* The activity of SS is highly correlated with sucrose content so that changes in SS activity may largely explain the reduced sucrose content in *G. uralensis* under S condition and the increased sucrose content in *G. uralensis* under S + G2 condition. In sucrose decomposition, salt stress downregulated the gene *Glyur000064s00005640*-encoding INV by transcriptomic and qRT-PCR analysis, and salt stress also decreased NI activity that inhibited the hydrolysis of sucrose, which indicated that salt stress inhibited the hydrolysis of sucrose mainly by regulating NI activity resulted from downregulation of the gene *Glyur000064s00005640.* However, G2 downregulated the gene *Glyur000064s00005640*-encoding INV by qRT-PCR analysis, but G2 increased NI and AI activities in *G. uralensis* under salt stress that promoted the hydrolysis of sucrose, which indicated that gene *Glyur000064s00005640* is not the key gene that controlled NI and AI activities. Moreover, G2 upregulated the gene *Glyur000005s00001105*-encoding α-glucosidase by transcriptomic and qRT-PCR analyses, which suggested that G2 promoted the hydrolysis of sucrose under salt stress. Owing to sucrose accumulation under the S + G2 condition, the cell osmotic potential decreased. In order to avoid further water loss of leaves, G2 promoted sucrose hydrolysis and increased the accumulation of reducing sugar in *G. uralensis* under salt stress not only by upregulating invertase but also by upregulating α-glucosidase, thus could help *G. uralensis* produce more energy to cope with salt stress by facilitating sucrose hydrolysis. Correlation analysis results showed that sucrose content was positively correlated with NI activity, which indicated that the overmuch sucrose required higher NI activity for decomposition, which thus improved the activity of NI.

As for fructose, salt stress resulted in a significant decrease in fructose content, while G2 resulted in a significant increase in fructose content in *G. uralensis*. In fructose synthesis, invertases and α-glucosidase mentioned above can catalyze the cleavage of sucrose to glucose and fructose; therefore, these enzymes not only participate in sucrose hydrolysis but also contribute to the production of fructose. In the present study, salt stress downregulated the gene *Glyur000064s00005640*-encoding INV by transcriptomic and qRT-PCR analyses, and salt stress also decreased NI activity that inhibited the production of fructose, which indicated that salt stress inhibited the production of fructose mainly by regulating NI activity caused by downregulation of the gene *Glyur000064s00005640.* G2 downregulated the gene *Glyur000064s00005640*-encoding INV by qRT-PCR analysis, but G2 increased NI and AI activities in *G. uralensis* under salt stress that promoted the production of fructose, which indicated that gene *Glyur000064s00005640* is not the key gene that regulated NI and AI activities under the S + G2 condition, and the potential reason needs further studies. Moreover, G2 upregulated the gene *Glyur000005s00001105*-encoding α-glucosidase by transcriptomic and qRT-PCR analyses, which further verified G2 promoted the production of fructose under salt stress not only by upregulating invertase but also by upregulating α-glucosidase. In fructose decomposition, HK, as a key enzyme in EMP, can phosphorylate glucose and fructose and induce leaf senescence (Sun et al., 2021). In the present study, salt stress upregulated gene *Glyur000324s00015431*-encoding HK by transcriptomic and qRT-PCR analyses, but salt stress decreased HK activity that inhibited the phosphorylation of fructose. However, G2 downregulated gene *Glyur000324s00015431*-encoding HK by qRT-PCR analysis, but G2 increased HK activity in *G. uralensis* under salt stress that promoted the phosphorylation of fructose and thus provided sufficient substrates for EMP. These results suggested that the gene *Glyur000324s00015431* might not be the pivotal gene that controlled HK activity, and the reason needs further studies. The activity of NI is highly correlated with fructose content so that changes in NI activity may largely explain the reduced fructose content in *G. uralensis* under the S condition and the increased fructose content in *G. uralensis* under the S + G2 condition, and then the overmuch fructose content required higher HK activity to decompose, which thus improved the HK activity.

In the starch and sucrose metabolism pathway, in addition to the genes mentioned above, the genes-encoding β-glucosidase and TPS in *G. uralensis* were affected by salt or G2. β-Glucosidase can catalyze the hydrolysis of the β-glycosidic linkage from the nonreducing end of isoflavone glucosides, disaccharides, oligosaccharides, aryl-glucosides, and alkyl-glucosides (Maitan-Alfenas et al., 2014). However, as a major component of the primary cell wall of many plant tissues, β-glucosidase may be involved in the production of signaling molecules through its specific hydrolytic activity, thus improving the plant’s salt tolerance (Mostek et al., 2016). In our study, salt stress downregulated the gene *Glyur000214s00016036*-encoding β-glucosidase by transcriptomic and qRT-PCR analyses, but upregulated the gene *Glyur000585s00027748*-encoding β-glucosidase by transcriptomic and qRT-PCR analyses and the gene *Glyur000779s00022830*-encoding β-glucosidase by transcriptomic analysis in *G. uralensis*, which implied that β-glucosidase could involve in the starch and sucrose metabolism pathway through affecting hydrolytic activity. G2 downregulated the gene *Glyur000214s00016036*-encoding β-glucosidase by qRT-PCR analysis but had no significant effect on genes-encoding β-glucosidase by transcriptomic analysis in *G. uralensis* under salt stress; thus, the effect of β-glucosidase by G2 on *G. uralensis* remains to be further studied. TPS can catalyze UDP-glucose and glucose-6-phosphate to form trehalose 6-phosphate (Tre6P), and then trehalose 6-phosphate phosphatase (TPP) can catalyze trehalose from Tre6P (Wang P. et al., 2020). Overexpression of the TPS gene in plants can enhance the ability of plant cells to scavenge ROS, promote the low accumulation of O_2_ and H_2_O_2_, thereby reducing cell death and enhancing salt tolerance and permeability of plants (Wang P. et al., 2020). In the present study, salt stress downregulated the genes (*Glyur000018s00003759* and *Glyur000231s00022077*)-encoding TPS, while G2 upregulated the gene *Glyur000018s00003759*-encoding TPS under salt stress in *G. uralensis* that may alleviate the oxidative stress caused by salt stress, thus improving the salt tolerance of *G. uralensis.*

Normal carbohydrate metabolism persists when plants grow unstressed conditions; in such a case, energy is stored in the form of starch, and the accumulation of TSS usually remains very limited. TSS concentrations are required for osmo-protection and carbon storage in plants subjected to salt stress (Neera and Amrit, 2018).

### Effect of G2 on the EMP-Tricarboxylic Acid Cycle in *Glycyrrhiza uralensis* Under Salt Stress

The EMP pathway-TCA cycle plays an important role in the aerobic respiration of plants, providing ATP, reductants, and metabolites needed for plant growth and development (Jiang, 2011). Sucrose and starch are sources of the EMP substrate fructose (Li et al., 2020), and they store chemical energy that can be released in the form of ATP through the EMP pathway-TCA cycle (Bandehagh and Taylor, 2020), and our study found that G2 increased the sucrose, starch, and fructose contents, which result in the EMP pathway being promoted from the substrate and energy level. In plants, HK, PFK, and PK are crucial for regulating the EMP pathway. HK is supposed to act as a sugar sensor and/or interact with other enzymes directly in supplying metabolic pathways (Kim et al., 2013). PFK is the rate-limiting enzyme in the EMP pathway because of its low catalytic efficiency and irreversible catalytic reaction (Wen et al., 2020). PK, a transferase involved in the final step of EMP, catalyzes PEP and adenosine diphosphate (ADP), resulting in one molecule of PA and one molecule of ATP. In our study, salt stress upregulated the gene *Glyur000324s00015431*-encoding HK by transcriptomic and qRT-PCR analyses, but decreased HK activity that inhibited the EMP process at the substrate level. However, G2 downregulated gene *Glyur000324s00015431*-encoding HK by qRT-PCR analysis, but increased HK activity in *G. uralensis* under salt stress. These results implied that the gene *Glyur000324s00015431* might not be the pivotal gene that regulates HK activity. The increase of HK activity by G2 could not only promote the EMP process at the substrate level but also help to alleviate the oxidative stress caused by the decrease of HK activity in *G. uralensis* under salt stress (Poór et al., 2019). Salt stress upregulated the gene *Glyur000219s00011582*-encoding PFK in transcriptomic and qRT-PCR results and also increased PFK and PK activity in *G. uralensis*, resulting in increased respiration in response to elevated energy requirements of *G. uralensis* from exposure to salt (Jacoby et al., 2011), which suggested that salt stress produced more energy by regulating PK and PFK resulted from upregulation of the gene *Glyur000219s00011582*. The increased PFK and PK activities may contribute to the acceleration of glucose catabolism, further produce more energy to remedy the inadequacy of energy caused by insufficient carbon sources, and, finally, maintain the *G. uralensis*’s normal life activities under salt stress, which is supported by the previous studies in tomato (Poór et al., 2019) and mangrove tree (Suzuki et al., 2005). However, G2 downregulated the gene *Glyur000219s00011582*-encoding PFK by qRT-PCR analysis and also decreased PFK and PK activities in *G. uralensis* under salt stress, which suggested that the EMP process was slowed down by regulating PK and PFK resulted from downregulation of the gene *Glyur000219s00011582*. In which case, the decrease of PFK and PK activities may be due to *G. uralensis*-elevated energy requirements from exposure to salt had been alleviated by G2. Additionally, an induced CA content can enhance the inhibitory effect of ATP and thus inhibit PFK activity (Sadka et al., 2019), which may help to explain why the process of EMP was slowed down.

In addition to the genes-encoding HK and PFK mentioned above, PDC, ADH, and ALDH-related genes in the EMP pathway were affected by salt or G2 in *G. uralensis*. There are three enzymes in the anaerobic metabolic pathway, PDC, ADH, and lactate dehydrogenase (LDH), in which PDC converted PA to acetaldehyde that was transformed to ethanol by ADH. Acetaldehyde and acetaldehyde are harmful to plant cells (Pan et al., 2019). ALDH further oxidizes acetaldehyde into carboxylic acids that can help cope with salt stress (Tagnon and Simeon, 2017). In our study, all of the DEGs-encoding PDC, ADH, and ALDH were upregulated in S vs. CK comparison, indicating that salt stress could firstly cause to produce more acetaldehyde and ethanol in *G. uralensis* by upregulating genes-encoding PDC and ADH and thus injure plant cells; in such a case, ALDH was upregulated timely for oxidizing excessive acetaldehyde into carboxylic acids that help *G. uralensis* cope with salt stress. However, only the gene-encoding PDC was downregulated in S + G2 vs. S comparison in *G. uralensis*, suggesting that G2 could protect plant cells from the impairment caused by toxic acetaldehyde and ethanol in *G. uralensis* under salt stress.

Pyruvate as the final product of the EMP pathway not only plays an important role in improving the salt tolerance of plants (Wu et al., 2013) but also links the EMP pathway with the TCA cycle. In our study, salt stress caused the PA production enzyme (PK) activity to increase and the PA decomposition enzyme (PDH) activity to decrease, but salt stress had no significant effect on PA content. This phenomenon may be due to the anaplerotic function of phosphoenolpyruvate carboxylase (PEPC) that replenished the TCA cycle with intermediates by catalyzing PA to produce oxaloacetate (Figures 4, 8), which finally ensured the normal progress of the TCA in *G. uralensis* under salt stress, which is supported by the results in peanut (*Arachis hypogaea* L.) (Pan et al., 2017). However, G2 caused the PA production enzyme (PK) activity to decrease and the PA decomposition enzyme (PDH) activity to increase, but had no significant effect on PA content in *G. uralensis* under salt stress, which may be due to G2 alleviated the inhibition to the TCA cycle from exposure to salt, and the TCA cycle can proceed normally, and the replenishment mechanism of PEPC was weakened, thereby reducing the consumption of PA in *G. uralensis* under salt stress.

The TCA cycle as the most effective way for an organism to obtain energy from the oxidation of sugar and other substances (Yu et al., 2021) can produce the largest amount of energy to plant and provide ATP and reductants for adaptive processes, such as ion exclusion, compatible solute synthesis, and ROS detoxification under salt stress (Che-Othman et al., 2019). Firstly, PDH as a bridge linking the EMP pathway to the TCA cycle can catalyze the oxidative decarboxylation of PA into acetyl CoA, which is the main input of energy in several steps of the TCA cycle (Saha et al., 2012). PDH complex is a complex consisting of three components: pdhB, dihydrolipoyl acetyltransferase (pdhC), and pdhD. In our study, salt stress upregulated the genes *Glyur000278s00017282* and *Glyur000082s00007586*-encoding pdhD and pdhB by transcriptomic and qRT-PCR analyses, but decreased PDH activity in *G. uralensis* that possibly inhibits energy production. However, G2 downregulated the genes *Glyur000278s00017282* and *Glyur000082s00007586*-encoding pdhD and pdhB by qRT-PCR analysis, but increased PDH activity in *G. uralensis* under salt stress that could provide energy for subsequent life activities of *G. uralensis*. These results indicated that the process of oxidative decarboxylation of pyruvate to acetyl-coA is complex, and the key genes regulating PDH need to be further identified. CA is the first organic acid generated in the TCA cycle, and the increase of CA content could help to resist the ionic stress in the plant (Torre-González et al., 2017). In our study, salt stress significantly decreased the CA content, while G2 significantly increased the CA content under salt stress, which potentially indicates an increased capacity for acetyl-CoA-dependent synthesis of organic acid resulted from the increase of PDH activity in *G. uralensis* by G2. These results are supported by Che-Othman et al. (2019) for wheat. Moreover, CA can inhibit PFK activity by enhancing the inhibitory effect of ATP under S + G2 treatment, thus slowing down the EMP process in *G. uralensis*, which is strongly supported by the results from Sadka et al. (2019). The next three important steps in the TCA cycle are conversion of isocitrate to α-ketoglutarate catalyzed by ICDH, succinate to fumarate catalyzed by SDH with the generation of energy in the form of FADH_2_, and malate to oxaloacetate catalyzed by MDH (Saha et al., 2012). Specifically, ICDH regulates nitrogen assimilation by maintaining the 2-oxoglutarate level (Bustamante et al., 2019), thereby linking C and N metabolism, and relates to plant antioxidants (Liu et al., 2010). In our study, G2 increased ICDH activity in *G. uralensis* under salt stress, which could affect C and N metabolism and antioxidant system. The accumulated CA under the S + G2 condition resulted from the increase of PDH activity needs higher ICDH to catalyze, thereby the ICDH activity was increased under the S + G2 condition. SDH plays a key role in mitochondrial metabolism both as a member of the electron transport chain and the TCA cycle (Araújo et al., 2011). Salt stress decreased SDH activity in *G. uralensis*, which could attribute to chloride that has a strong inhibitory effect on SDH activity, which is supported by Nunes-Nesi et al. (2013). However, G2 decreased SDH activity in *G. uralensis* under salt stress, which may attribute to the mitigation of chlorine toxicity. The TCA cycle is completed by MDH, which, as a salt-sensitive enzyme, is thought to play a protective role by counteracting the damaging effect of salt by increasing the malate through conformational change (Saha et al., 2012). Salt stress enhanced MDH activity in *G. uralensis*, and the more active MDH could offset the damaging effect of salinity by conformational changes, thus protecting *G. uralensis* from salt stress.

It was concluded that the G2 slowed-down EMP pathway may be attributed to the following reasons. Firstly, G2 alleviated the elevated energy requirements from exposure to salt, thereby decreased PFK and PK activities in *G. uralensis* under salt stress. Secondly, increased CA content inhibited PFK activity by enhancing the inhibitory effect of ATP under the S + G2 condition. Moreover, G2 increased HK activity, which may inhibit the production of ROS and increased ICDH activity, which may affect the production of plant antioxidants, thus possibly improving salt tolerance of *G. uralensis* by affecting the antioxidant system. PDH, as a key enzyme for major energy regulation in the TCA cycle, was promoted by G2 under salt stress in *G. uralensis*, which activated the TCA cycle, resulting in the production of large amounts of intermediates and energy to support *G. uralensis* growth under salt stress.

## Conclusion

G2 improved Chl content in *G. uralensis* under salt stress, which may lead to highly efficient photosynthesis in terms of light reaction. Second, it increased carbohydrate contents in *G. uralensis* due to balanced regulation of related enzymes, thus increased *G. uralensis* growth and yield under salt stress, and the increase of TSS content induced by G2 plays a protective role in osmoregulation in *G. uralensis* seedlings under salt stress. Third, it alleviated the elevated energy requirements from exposure to salt, thereby slowing down the EMP process in *G. uralensis* under salt stress. Fourth, it provided energy for the subsequent life activities of *G. uralensis* under salt stress through regulating the TCA cycle. Therefore, G2 can effectively regulate and accumulate carbohydrate, improve salt tolerance, and thereby promote the growth of *G. uralensis* seedlings under salt stress.

## Data Availability Statement

The data presented in the study are deposited in the GEO repository, accession number GSE187003 (https://www.ncbi.nlm.nih.gov/geo/query/acc.cgi?acc=GSE187003).

## Author Contributions

XX performed the experiment and wrote the manuscript. QW made the tables. XM modified the language. DL modified the details and made the figures. XZ provided the ideas and revised the manuscript. ZG revised the manuscript. All authors contributed to the article and approved the submitted version.

## Conflict of Interest

The authors declare that the research was conducted in the absence of any commercial or financial relationships that could be construed as a potential conflict of interest.

## Publisher’s Note

All claims expressed in this article are solely those of the authors and do not necessarily represent those of their affiliated organizations, or those of the publisher, the editors and the reviewers. Any product that may be evaluated in this article, or claim that may be made by its manufacturer, is not guaranteed or endorsed by the publisher.

## Supplementary Material

Supplementary Figure 1 A correlation line graph between carbohydrates and photosynthetic pigment contents, carbohydrates transformation, and EMP pathway-TCA cycle indexes in *G. uralensis* seedlings. **(A)** The causal relationship between photosynthetic pigment contents and carbohydrates in *G. uralensis* seedlings. **(B)** The causal relationship between starch and sucrose-related enzymes and carbohydrates in *G. uralensis* seedlings. **(C)** The causal relationship between EMP pathway-TCA cycle-related enzymes and intermediates and carbohydrates in *G. uralensis* seedlings. The solid and dotted lines indicate the positive and negative effects, respectively. CK, control group; S, salt stress group; CK + G2, control combined with the G2 group; S + G2, salt stress combined with the G2 group. The different letters within the different treatments in the same parameter indicate the significant difference at the 0.05 level. Values are means ± SE (*n* = 3). Chl *a*, chlorophyll *a*; Chl *b*, chlorophyll *b*; Car, carotenoids; AGP, ADP-glucose pyrophosphorylase; GBSS, granule-bound starch synthase; SSS, soluble starch synthase; AM, α-amylase; BM, β-amylase; SPS, sucrose-phosphate synthase; SS, sucrose synthase; AI, acid invertase; NI, neutral invertase; HK, hexokinase; PFK, phosphofructokinase; PK, pyruvate kinase; PDH, pyruvate dehydrogenase; ICDH, isocitrate dehydrogenase; SDH, succinate dehydrogenase; MDH, malate dehydrogenase; Sta, starch; Fru, fructose; Suc, sucrose.

Supplementary Figure 2 Principal component analysis of enzyme activity and gene expression in photosynthesis, starch and sucrose metabolism, and EMP and TCA cycle indexes of *G. uralensis* seedlings under four treatments (CK, CK + G2, S, and S + G2). **(A)** Ordering distribution and interpretation of the 12 principal components related to enzyme activity. **(B)** The contribution of the variables related to photosynthetic pigments content, starch and sucrose metabolism, and EMP and TCA cycle indexes to the principal components. **(C)** Ordering distribution and interpretation of the 12 principal components related to gene expression. **(D)** The contribution of the variables related to carbon fixation in the photosynthetic organisms pathway, starch and sucrose metabolism, and EMP and TCA cycle indexes to the principal components. The depth of the color of the arrow line represents the degree of contribution. The angle of the arrow line represents correlations. CK, control group; S, salt stress group; CK + G2, control combined with the G2 group; S + G2, salt stress combined with the G2 group.

Supplementary Figure 3 Expression of carbohydrate metabolism-related genes (RT-PCR) in *G. uralensis* in four treatments (CK, CK + G2, S, and S + G2). CK, control group; S, salt stress group; CK + G2, control combined with the G2 group; S + G2, salt stress combined with the G2 group.

## References

[B1] Ahmadi F. I. Karimi K. Struik P. C. (2018). Effect of exogenous application of methyl jasmonate on physiological and biochemical characteristics of *Brassica napus* L. cv. Talaye under salinity stress. *South Afr. J. Bot.* 115 5–11. 10.1016/j.sajb.2017.11.018

[B2] Ali S. Charles T. C. Glick B. R. (2014). Amelioration of high salinity stress damage by plant growth-promoting bacterial endophytes that contain ACC deaminase. *Plant Physiol. Biochem.* 80 160–167. 24769617 10.1016/j.plaphy.2014.04.003

[B3] Aliche E. B. Theeuwen T. P. J. M. Oortwijn M. Visser R. G. F. van der Linden C. G. (2019). Carbon partitioning mechanisms in POTATO under drought stress. *Plant Physiol. Biochem.* 146 211–219. 10.1016/j.plaphy.2019.11.019 31756607

[B4] Araújo W. L. Nunes-Nesi A. Osorio S. Usadel B. Fuentes D. Nagy R. (2011). Antisense inhibition of the iron-sulphur subunit of succinate dehydrogenase enhances photosynthesis and growth in tomato via an organic acid-mediated effect on stomatal aperture. *Plant Cell* 23 600–627. 10.1105/tpc.110.081224 21307286 PMC3077794

[B5] Bandehagh A. Taylor N. L. (2020). Can alternative metabolic pathways and shunts overcome salinity induced inhibition of central carbon metabolism in crops? *Front. Plant Sci.* 11:1072. 10.3389/fpls.2020.01072 32849676 PMC7417600

[B6] Baslam M. Mitsui T. Sueyoshi K. Ohyama T. (2020). Recent advances in carbon and nitrogen metabolism in C3 plants. *Int. J. Mol. Sci.* 22:318. 10.3390/ijms22010318 33396811 PMC7795015

[B7] Bilska-Kos A. Mytych J. Suski S. Magoń J. Zebrowski J. (2020). Sucrose phosphate synthase (SPS), sucrose synthase (SUS) and their products in the leaves of Miscanthus × giganteus and *Zea mays* at low temperature. *Planta* 252:23. 10.1007/s00425-020-03421-2 32676847 PMC7366575

[B8] Borghesi E. González-Miret M. L. Escudero-Gilete M. L. Malorgio F. Heredia F. J. Meléndez-Martínez A. J. (2011). Effects of salinity stress on carotenoids, anthocyanins, and color of diverse tomato genotypes. *J. Agric. Food Chem.* 59 11676–11682. 10.1021/jf2021623 21923118

[B9] Brahimova U. Kumari P. Yadav S. Rastogi A. Brestic M. (2021). Progress in understanding salt stress response in plants using biotechnological tools. *J. Biotechnol.* 329 180–191. 10.1016/j.jbiotec.2021.02.007 33610656

[B10] Bustamante M. A. Nogués I. Jones S. Allison G. G. (2019). The effect of anaerobic digestate derived composts on the metabolite composition and thermal behaviour of rosemary. *Sci. Rep.* 9:6489. 10.1038/s41598-019-42725-6 PMC6482180 31019202

[B11] Chaves M. M. Flexas J. Pinheiro C. (2008). Photosynthesis under drought and salt stress: regulation mechanisms from whole plant to cell. *Ann. Bot.* 103 551–560. 10.1093/aob/mcn125 18662937 PMC2707345

[B12] Che-Othman M. H. Jacoby R. P. Millar A. H. Taylor N. L. (2019). Wheat mitochondrial respiration shifts from the TCA cycle to the GABA shunt under salt stress. *New Phytol.* 225, 1166–1180. 10.1111/nph.15713 30688365

[B13] Chintakovid N. Maipoka M. Phaonakrop N. Mickelbart M. V. Roytrakul S. Chadchawan S. (2017). Proteomic analysis of drought-responsive proteins in rice reveals photosynthesis-related adaptations to drought stress. *Acta Physiol. Plant.* 39:240. 10.1007/s11738-017-25

[B14] Devi R. Munjral N. Gupta A. K. Kaur N. (2007). Cadmium induced changes in carbohydrate status and enzymes of carbohydrate metabolism, glycolysis and pentose phosphate pathway in pea. *Environ. Exp. Bot.* 61 167–174. 10.1016/j.envexpbot.2007.05.006

[B15] El Atta H. A. Aref I. M. Ahmed A. I. (2016). Seed size effects on the response of seedlings of *Acacia asak* (Forssk.) Willd. to water stress. *Pakistan J. Bot.* 48 439–446.

[B16] El-Esawi M. A. Alaraidh I. A. Alsahli A. A. Alamri S. A. Ali H. M. Alayafi A. A. (2018). Bacillus firmus (SW5) augments salt tolerance in soybean (*Glycine max* L.) by modulating root system architecture, antioxidant defense systems and stress-responsive genes expression. *Plant Physiol. Biochem.* 132 375–384. 10.1016/j.plaphy.2018.09.026 30268029

[B17] Fan D. Subramanian S. Smith D. L. (2020). Plant endophytes promote growth and alleviate salt stress in Arabidopsis thaliana. *Sci. Rep.* 10 12740. 10.1038/s41598-020-69713-5 32728116 PMC7391687

[B18] Frukh A. Siddiqi T. O. Iqbal M. Khan R. Ahmad A. (2019). Modulation in growth, biochemical attributes and proteome profile of rice cultivars under salt stress. *Plant Physiol. Biochem.* 146 55–70. 10.1016/j.plaphy.2019.11.011 31733605

[B19] Gao J. F. (2006). *Experimental Guide For Plant Physiology.* Beijing: Higher Education Press.

[B20] Ghaffari M. R. Ghabooli M. Khatabi B. Hajirezaei M. R. Schweizer P. Salekdeh G. H. (2016). Metabolic and transcriptional response of central metabolism affected by root endophytic fungus piriformospora indica under salinity in barley. *Plant Mol. Biol.* 90 699–717. 10.1007/s11103-016-0461-z 26951140

[B21] Gil R. Boscaiu M. Lull C. Bautista I. Lidón A. Vicente O. (2013). Are soluble carbohydrates ecologically relevant for salt tolerance in halophytes? *Funct. Plant Biol.* 40 805–818. 10.1071/fp12359 32481152

[B22] Gururani M. A. Venkatesh J. Tran L. P. (2015). Regulation of photosynthesis during abiotic stress-induced photoinhibition. *Mol. Plant* 8 1304–1320. 10.1016/j.molp.2015.05.005 25997389

[B23] Hou L. Li X. He X. Zuo Y. Zhang D. Zhao L. (2021). Effect of dark septate endophytes on plant performance of *Artemisia ordosica* and associated soil microbial functional group abundance under salt stress. *Appl. Soil Ecol.* 165:103998. 10.1016/j.apsoil.2021.103998

[B24] Huang C. J. Wei G. Jie Y. C. Xu J. J. Zhao S. Y. (2015). Responses of gas exchange, chlorophyll synthesis and ROS-scavenging systems to salinity stress in two ramie (*Boehmeria nivea* L.) cultivars. *Photosynthetica* 53 455–463. 10.1007/s11099-015-0127-0

[B25] Ibarra-Villarreal A. Gándara-Ledezma A. Godoy-Flores A. D. Herrera-Sepúlveda A. Díaz-Rodríguez A. M. Parra-Cota F. I (2021). Salt-tolerant Bacillus species as a promising strategy to mitigate the salinity stress in wheat (*Triticum turgidum* subsp. durum). *J. Arid Environ.* 186:104399. 10.1016/j.jaridenv.2020.104399

[B26] Ibrahim W. Ahmed I. M. Chen X. Wu F. (2017). Genotype-dependent alleviation effects of exogenous GSH on salinity stress in cotton is related to improvement in chlorophyll content, photosynthetic performance, and leaf/root ultrastructure. *Environ. Sci. Pollut. Res.* 24 9417–9427. 10.1007/s11356-017-8611-7 28233214

[B27] Jacoby R. P. Taylor N. L. Millar A. H. (2011). The role of mitochondrial respiration in salinity tolerance. *Trends Plant Sci.* 16, 614–623. 10.1016/j.tplants.2011.08.002 21903446

[B28] Jiang D. A. (2011). *Plant Physiology.* Beijing: Higher Education Press.

[B29] Keunen E. Peshev D. Vangronsveld J. Wim V. Cuypers A. (2013). Plant sugars are crucial players in the oxidative challenge during abiotic stress: extending the traditional concept. *Plant Cell Environ.* 36 1242–1255. 10.1111/pce.12061 23305614

[B30] Khan A. L. Hamayun M. Kim Y. H. Kang S. M. Lee I. J. (2011). Ameliorative symbiosis of endophyte (*Penicillium funiculosum* LHL06) under salt stress elevated plant growth of *Glycine max* L. *Plant Physiol. Bioch.* 49 852–861. 10.1016/j.plaphy.2011.03.005 21458283

[B31] Khoshbakht D. Ramin A. A. Baninasab B. (2015). Effects of sodium chloride stress on gas exchange, chlorophyll content and nutrient concentrations of nine citrus rootstocks. *Photosynthetica* 53 241–249.

[B32] Kim Y. M. Heinzel N. Giese J. O. Koeber J. Hajirezaei M. R. (2013). A dual role of tobacco hexokinase 1 in primary metabolism and sugar sensing. *Plant Cell Environ.* 36 1311–1327. 10.1111/pce.12060 23305564

[B33] Li L. Xing W. W. Shao Q. S. Shu S. Sun J. Guo S. R. (2015). The effects of grafting on glycolysis and the tricarboxylic acid cycle in Ca(NO3)2-stressed cucumber seedlings with pumpkin as rootstock. *Acta Physiol. Plant.* 37 259. 10.1007/s11738-015-1978-5

[B34] Li S. Li Y. Gao Y. He X. Li Q. (2020). Effects of CO2 enrichment on non-structural carbohydrate metabolism in leaves of cucumber seedlings under salt stress. *Sci. Hortic.* 265:109275. 10.1016/j.scienta.2020.109275

[B35] Li Y. P. Yu C. X. Qiao J. Zang Y. M. Xiang Y. Ren G. X. (2016). Effect of exogenous phytohormones treatment on glycyrrhizic acid accumulation and preliminary exploration of the chemical control network based on glycyrrhizic acid in root of *Glycyrrhiza uralensis*. *Rev. Bras. Farmacogn.* 26 490–496. 10.1016/j.bjp.2016.02.009

[B36] Liu J. Wu N. Wang H. Sun J. Peng B. Jiang P. (2016). Nitrogen addition affects chemical compositions of plant tissues, litter and soil organic matter. *Ecology* 97 1796–1806. 10.1890/15-1683.1 27859176

[B37] Liu Y. Shi Y. Song Y. Wang T. Yu L. (2010). Characterization of a stress-induced NADP-isocitrate dehydrogenase gene in maize confers salt tolerance in *Arabidopsis*. *J. Plant Biol.* 53 107–112. 10.1007/s12374-009-9091-1

[B38] Lloyd J. R. Kötting O. (2016). *Starch Biosynthesis And Degradation In Plants.* Hoboken, NJ: John Wiley & Sons, Ltd, 10.1002/9780470015902.a0020124.pub2

[B39] Lombardo V. A. Osorio S. Borsani J. Lauxmann M. A. Bustamante C. A. Budde C. O. (2011). Metabolic profiling during peach fruit development and ripening reveals the metabolic networks that underpin each developmental stage. *Plant Physiol.* 157 1696–1710. 10.1104/pp.111.186064 22021422 PMC3327199

[B40] Maitan-Alfenas G. P. Lage L. Almeida M. N. D. Visser E. M. Rezende S. T. D. Guimaraes V. M. (2014). Hydrolysis of soybean isoflavones by *Debaryomyces hansenii* UFV-1 immobilised cells and free β-glucosidase. *Food Chem.* 146 429–436. 10.1016/j.foodchem.2013.09.099 24176363

[B41] Moigne T. L. Crozet P. Lemaire S. D. Henri J. (2020). High-resolution crystal structure of chloroplastic ribose-5-phosphate isomerase from *Chlamydomonas reinhardtii*-an enzyme involved in the photosynthetic calvin-benson cycle. *Int. J. Mol. Sci.* 21:7787. 10.3390/ijms21207787 33096784 PMC7589169

[B42] Molina-Montenegro M. A. Acuña-Rodríguez I. S. Torres-Díaz C. Gundel P. E. Dreyer I. (2020). Antarctic root endophytes improve physiological performance and yield in crops under salt stress by enhanced energy production and Na+sequestration. *Sci. Rep.* 10:5819. 10.1038/s41598-020-62544-4 32242034 PMC7118072

[B43] Mostek A. Brner A. Weidner S. (2016). Comparative proteomic analysis of β-aminobutyric acid-mediated alleviation of salt stress in barley. *Plant Physiol. Biochem.* 99 150–161. 10.1016/j.plaphy.2015.12.007 26760953

[B44] Neera G. Amrit B. (2018). Salicylic acid improves arbuscular mycorrhizal symbiosis, and chickpea growth and yield by modulating carbohydrate metabolism under salt stress. *Mycorrhiza.* 28 727–746. 10.1007/s00572-018-0856-6 30043257

[B45] Nunes-Nesi A. Araújo W. L. Obata T. Fernie A. R. (2013). Regulation of the mitochondrial tricarboxylic acid cycle. *Curr. Opin. Plant Biol.* 16 335–343. 10.1016/j.pbi.2013.01.004 23462640

[B46] Pan D. L. Wang G. Wang T. Jia Z. H. Guo Z. R. Zhang J. Y. (2019). AdRAP2.3, a Novel Ethylene Response Factor VII from Actinidia deliciosa, enhances waterlogging resistance in transgenic tobacco through improving expression levels of PDC and ADHgenes. *Int. J. Mol. Sci.* 20:1189. 10.3390/ijms20051189 30857203 PMC6429156

[B47] Pan L. Zhang J. Chen N. Chen M. Wang M. Wan T. (2017). Molecular characterization and expression profiling of the phosphoenolpyruvate carboxylase genes in peanut (*Arachis hypogaea* L.). *Russian J. Plant Physiol.* 64 576–587. 10.1134/S1021443717040100

[B48] Poór P. Patyi G. Takács Z. Szekeres A. Tari I. (2019). Salicylic acid-induced ROS production by mitochondrial electron transport chain depends on the activity of mitochondrial hexokinases in tomato (*Solanum lycopersicum* L.). *J. Plant Res.* 132 273–283. 10.1007/s10265-019-01085-y 30758749 PMC7196940

[B49] Qiu T. Jiang L. L. Li S. Z. Yang Y. F. (2017). Small-scale habitat-specific variation and adaptive divergence of photosynthetic pigments in different alkali soils in reed identified by common garden and genetic tests. *Front. Plant Sci.* 7:2016. 10.3389/fpls.2016.02016 28111586 PMC5216671

[B50] Rabiei Z. Hosseini S. J. Pirdashti H. Hazrati S. (2020). Physiological and biochemical traits in coriander affected by plant growth-promoting rhizobacteria under salt stress. *Heliyon* 6:e05321. 10.1016/j.heliyon.2020.e05321 33145448 PMC7591739

[B51] Raessler M. Wissuwa B. Breul A. Unger W. Grimm T. (2010). Chromatographic analysis of major non-structural carbohydrates in several wood species – an analytical approach for higher accuracy of data. *Anal. Methods* 2:532. 10.1039/b9ay00193j

[B52] Sadka A. Shlizerman L. Kamara I. Blumwald E. (2019). Primary metabolism in citrus fruit as affected by its unique structure. *Front. Plant Sci.* 10:1167. 10.3389/fpls.2019.01167 31611894 PMC6775482

[B53] Saha P. Kunda P. Biswas A. K. (2012). Influence of sodium chloride on the regulation of krebs cycle intermediates and enzymes of respiratory chain in mungbean (*Vigna radiata* L. wilczek) seedlings – sciencedirect. *Plant Physiol. Biochem.* 60 214–222. 10.1016/j.plaphy.2012.08.008 23000814

[B54] Salimi F. Shekari F. Hamzei J. (2016). Methyl jasmonate improves salinity resistance in *German chamomile* (*Matricaria chamomilla* L.) by increasing activity of antioxidant enzymes. *Acta Physiol. Plant.* 38:1. 10.1007/s11738-015-2023-4

[B55] Scholes J. Bundock N. Wilde R. Rolfe S. (1996). The impact of reduced vacuolar invertase activity on the photosynthetic and carbohydrate metabolism of tomato. *Planta* 200 265–272. 10.1007/BF00208317

[B56] Sun L. Song F. Zhu X. Liu S. Liu F. Wang Y. (2021). Nano-zno alleviates drought stress via modulating the plant water use and carbohydrate metabolism in maize. *Arch. Agron. Soil Sci.* 67 245–259. 10.1080/03650340.2020.1723003

[B57] Suzuki M. Hashioka A. Mimura T. Ashihara H. (2005). Salt stress and glycolytic regulation in suspension-cultured cells of the mangrove tree *Bruguiera sexangula*. *Physiol. Plant.* 123 246–253. 10.1111/j.1399-3054.2005.00456.x

[B58] Tagnon M. D. Simeon K. O. (2017). Aldehyde dehydrogenases may modulate signaling by lipid peroxidation-derived bioactive aldehydes. *Plant Signal. Behav.* 12:e1387707. 10.1080/15592324.2017.1387707 28990846 PMC5703241

[B59] Tang Z. C. (1999). *Experimental Guide of Modern Plant Physiology.* Beijing: Science Press.

[B60] Torre-González A. D. L. Albacete A. Sánchez E. Blasco B. Ruiz J. M. (2017). Comparative study of the toxic effect of salinity in different genotypes of tomato plants: carboxylates metabolism. *Sci. Hortic.* 217 173–178. 10.1016/j.scienta.2017.01.045

[B61] Wang C. Chen L. Cai Z. Chen C. Mei Y. (2020). Comparative proteomic analysis reveals the molecular mechanisms underlying the accumulation difference of bioactive constituents in *Glycyrrhiza uralensis* Fisch under salt stress. *J. Agric. Food Chem.* 68 1480–1493. 10.1021/acs.jafc.9b04887 31899641

[B62] Wang P. Lei X. Lü J. Gao C. (2020). Overexpression of the thtps gene enhanced salt and osmotic stress tolerance in *Tamarix hispida*. *J. For. Res.* 10.1007/s11676-020-01224-5

[B63] Wellburn A. R. (1994). The spectral determination of chlorophyll a and chlorophyll b, as well as total carotenoids, using various solvents with spectrophotometers of different resolution. *J. Plant Physiol.* 144 307–313. 10.1016/S0176-1617(11)81192-2

[B64] Wen J. Wang W. Xu K. Ji D. Xu Y. Chen C. (2020). Comparative analysis of proteins involved in energy metabolism and protein processing in Pyropia haitanensis at different salinity levels. *Front. Mar. Sci.* 7:415. 10.3389/fmars.2020.00415

[B65] Win K. T. Tanaka F. Okazaki K. Ohwaki Y. (2018). The ACC deaminase expressing endophyte *Pseudomonas* spp. Enhances NaCl stress tolerance by reducing stress-related ethylene production, resulting in improved growth, photosynthetic performance, and ionic balance in tomato plants. *Plant Physiol. Biochem.* 127 599–607. 10.1016/j.plaphy.2018.04.038 29730579

[B66] Wu D. Cai S. Chen M. Ye L. Chen Z. Zhang H. (2013). Tissue metabolic responses to salt stress in wild and cultivated barley. *PLoS One* 8:e55431. 10.1371/journal.pone.0055431 23383190 PMC3561194

[B67] Wu P. Li X. Liu X. Xu X. Li L. (2020). Comparison of transcriptomic and proteomic analyses to construct a model for the promotion of starch synthesis by ABA in *Euryale ferox* Salisb. seeds. *Res. Square* [Preprint]. 10.21203/rs.3.rs-18154/v1

[B68] Yang Z. Li J. L. Liu L. N. Xie Q. Sui N. (2020). Photosynthetic regulation under salt stress and salt-tolerance mechanism of sweet sorghum. *Front. Plant Sci.* 10:1722. 10.3389/fpls.2019.01722 PMC6974683 32010174

[B69] Yu H. Yuan Y. Wang S. Wu G. Xu H. Wei J. (2021). Interspecies evolution and networks investigation of the auxin response protein (AUX/IAA) family reveals the adaptation mechanisms of halophytes crops in nitrogen starvation agroecological environments. *Agriculture* 11:780. 10.3390/agriculture11080780

[B70] Zhang W. Yu X. Li M. Lang D. Zhang X. Xie Z. (2018). Silicon promotes growth and root yield of Glycyrrhiza uralensis under salt and drought stresses through enhancing osmotic adjustment and regulating antioxidant metabolism. *Crop Prot.* 107 1–11. 10.1016/j.cropro.2018.01.005

[B71] Zhong M. Yuan Y. Shu S. Sun J. Guo S. Yuan R. (2016). Effects of exogenous putrescine on glycolysis and Krebs cycle metabolism in cucumber leaves subjected to salt stress. *Plant Growth Regul.* 79 319–330.

[B72] Zhou X. Lindsay H. Robinson M. D. (2014). Robustly detecting differential expression in RNA sequencing data using observation weights. *Nucleic Acids Res.* 42:e91. 10.1093/nar/gku310 24753412 PMC4066750

[B73] Zhu Y. Guo J. Feng R. Jia J. Han W. Gong H. (2016). The regulatory role of silicon on carbohydrate metabolism in *Cucumis sativus* L. under salt stress. *Plant Soil* 406 231–249. 10.1007/s11104-016-2877-2

