# Supplementary material for: Physiological Biochemistry-Combined Transcriptomic Analysis Reveals Mechanism of Bacillus cereus G2 Improved Salt-Stress Tolerance of Glycyrrhiza uralensis Fisch. Seedlings by Balancing Carbohydrate Metabolism
Source: Front Plant Sci. 2022 Jan 4;12:712363. doi: 10.3389/fpls.2021.712363 (PMC8764457; doi:10.3389/fpls.2021.712363)
Supplement: Supplementary file 1 [file Table_1.DOCX]

**Table S1 Names and sequences of the primers used for the amplification of the genes in the carbohydrate metabolism pathway in *G. uralensis*.**

| **Enzyme name** | **Gene ID/name** | **Primer F** | **Primer R** |
| --- | --- | --- | --- |
| β-glucosidase | *Glyur000585s00027748* | GCTGAGTCTGTGGTGCCTGAA | GCCATACCCTCTACTTGAAACGC |
|  | *Glyur000214s00016036* | TTCCTACCCCACCACTAATGAGAT | CTCTCACATCTGCTCCTTTCCTT |
| INV, sacA | *Glyur000064s00005640* | ATACCCTTCAAACCCTGTCCTG | GCATTCCCTCTACACGCTCATA |
| α-glucosidase | *Glyur000005s00001105* | TACCACTCCTACTCCACTCCCACT | CAAAGACTTGCCTGTTGTGGG |
| BM | *Glyur000067s00006388* | AAGGTGCTTGCTCCATCGG | GCTCAGCATAGGCTTCCCAGT |
| SS | *Glyur001957s00039090* | TTTGTTGCTTTGGCTGTTCG | TTAGCAGAAAGATGGCGGTTG |
| HK | *Glyur000324s00015431* | CTCGCATCTGAAGGTGGCA | ACACGAAGGACACGGAAGTTTG |
| PFK, pfkA | *Glyur000219s00011582* | CAGAGGAAGTGTATGCTTGTGTTG | CCTCTATTCCGAGAACTCTGTGC |
| pdhB, PDHB | *Glyur000278s00017282* | CCTGCGTGAAGGTTTGGAAG | TGTGAAGGCGTTCTCAGCAAT |
| pdhD, DLD, lpd | *Glyur000082s00007586* | CTCAACCGCCACAATCAACAG | TTCCCGCATACGACCACTAAC |
|  | *β-actin* | GAATTGCGTGTTGCTCCTG | TGTACGACCACTGGCATAAAGA |
