# Supplementary figures and images for: Physiological Biochemistry-Combined Transcriptomic Analysis Reveals Mechanism of Bacillus cereus G2 Improved Salt-Stress Tolerance of Glycyrrhiza uralensis Fisch. Seedlings by Balancing Carbohydrate Metabolism
Source: Front Plant Sci. 2022 Jan 4;12:712363. doi: 10.3389/fpls.2021.712363 (PMC8764457; doi:10.3389/fpls.2021.712363)

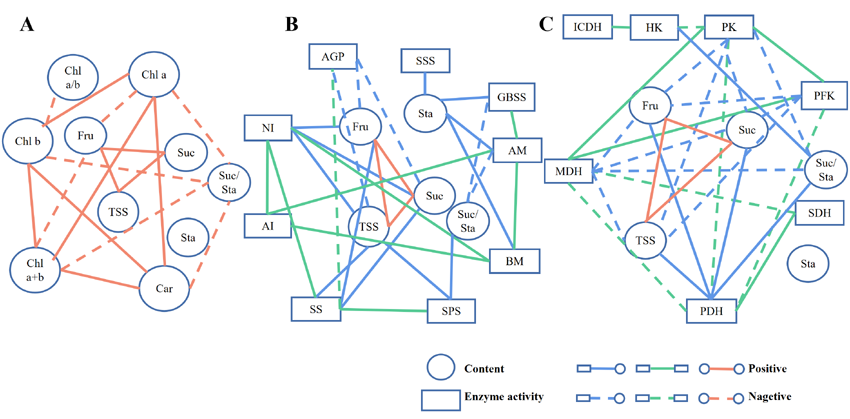

Supplement: Supplementary Figure 1 — A correlation line graph between carbohydrates and photosynthetic pigment contents, carbohydrates transformation, and EMP pathway-TCA cycle indexes in G. uralensis seedlings. (A) The causal relationship between photosynthetic pigment contents and carbohydrates in G. uralensis seedlings. (B) The causal relationship between starch and sucrose-related enzymes and carbohydrates in G. uralensis seedlings. (C) The causal relationship between EMP pathway-TCA cycle-related enzymes and intermediates and carbohydrates in G. uralensis seedlings. The solid and dotted lines indicate the positive and negative effects, respectively. CK, control group; S, salt stress group; CK + G2, control combined with the G2 group; S + G2, salt stress combined with the G2 group. The different letters within the different treatments in the same parameter indicate the significant difference at the 0.05 level. Values are means ± SE (n = 3). Chl a, chlorophyll a; Chl b, chlorophyll b; Car, carotenoids; AGP, ADP-glucose pyrophosphorylase; GBSS, granule-bound starch synthase; SSS, soluble starch synthase; AM, α-amylase; BM, β-amylase; SPS, sucrose-phosphate synthase; SS, sucrose synthase; AI, acid invertase; NI, neutral invertase; HK, hexokinase; PFK, phosphofructokinase; PK, pyruvate kinase; PDH, pyruvate dehydrogenase; ICDH, isocitrate dehydrogenase; SDH, succinate dehydrogenase; MDH, malate dehydrogenase; Sta, starch; Fru, fructose; Suc, sucrose. [file Image_1.TIF]

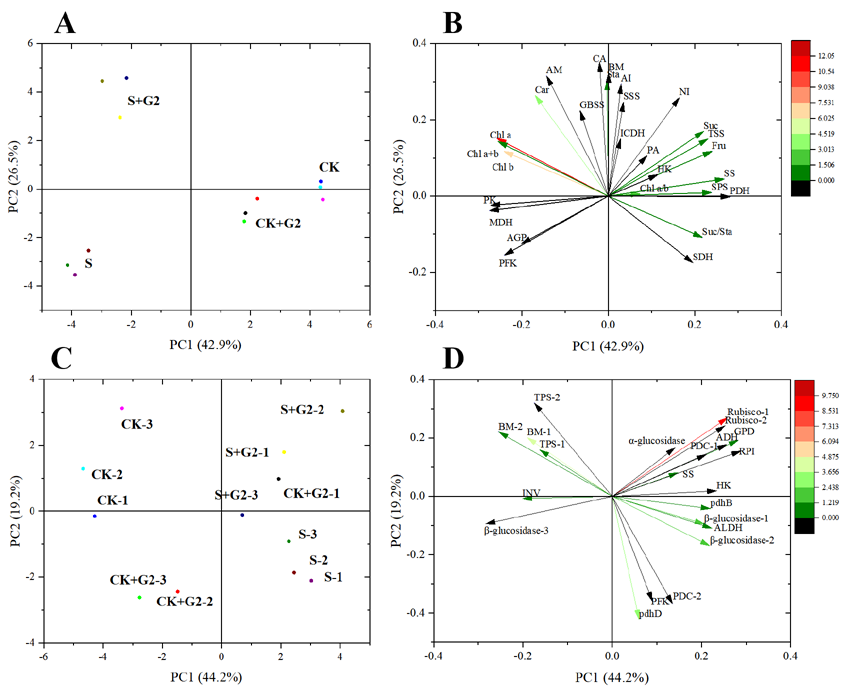

Supplement: Supplementary Figure 2 — Principal component analysis of enzyme activity and gene expression in photosynthesis, starch and sucrose metabolism, and EMP and TCA cycle indexes of G. uralensis seedlings under four treatments (CK, CK + G2, S, and S + G2). (A) Ordering distribution and interpretation of the 12 principal components related to enzyme activity. (B) The contribution of the variables related to photosynthetic pigments content, starch and sucrose metabolism, and EMP and TCA cycle indexes to the principal components. (C) Ordering distribution and interpretation of the 12 principal components related to gene expression. (D) The contribution of the variables related to carbon fixation in the photosynthetic organisms pathway, starch and sucrose metabolism, and EMP and TCA cycle indexes to the principal components. The depth of the color of the arrow line represents the degree of contribution. The angle of the arrow line represents correlations. CK, control group; S, salt stress group; CK + G2, control combined with the G2 group; S + G2, salt stress combined with the G2 group. [file Image_2.TIF]

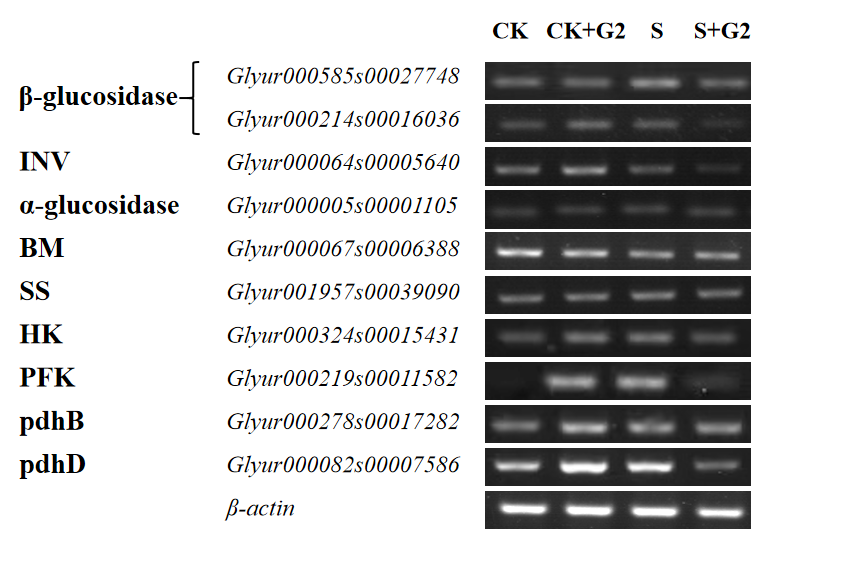

Supplement: Supplementary Figure 3 — Expression of carbohydrate metabolism-related genes (RT-PCR) in G. uralensis in four treatments (CK, CK + G2, S, and S + G2). CK, control group; S, salt stress group; CK + G2, control combined with the G2 group; S + G2, salt stress combined with the G2 group. [file Image_3.TIF]
